# Supplementary figures and images for: 5′ UTR length shapes alternative N-terminal protein isoforms across cancers and in rare disease
Source: EMBO Rep. 2026 Apr 13;27(10):2823–43. doi: 10.1038/s44319-026-00776-7 (PMC13219423; doi:10.1038/s44319-026-00776-7)

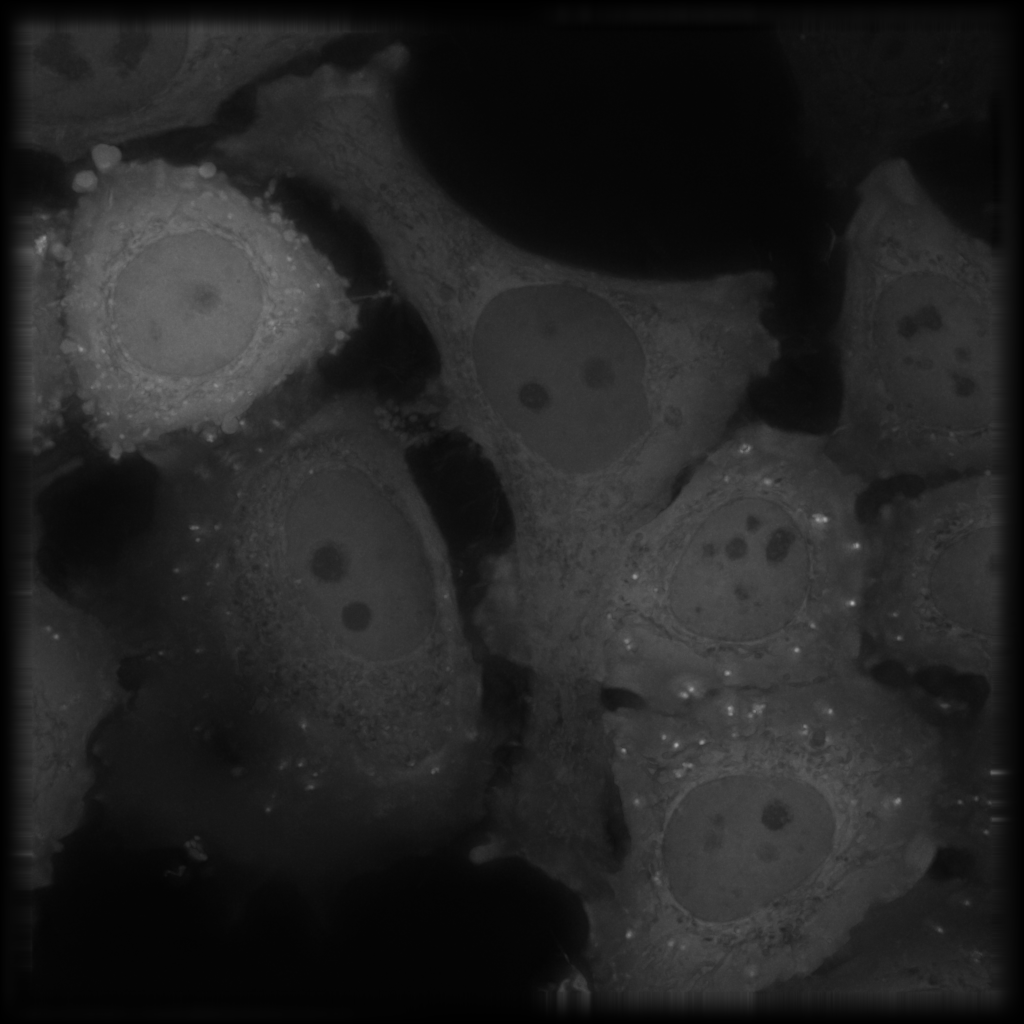

Supplement: Supplementary file 6 — Source data Fig. 1 [file 44319_2026_776_MOESM6_ESM.zip › Figure1/Figure1B_right.tif]

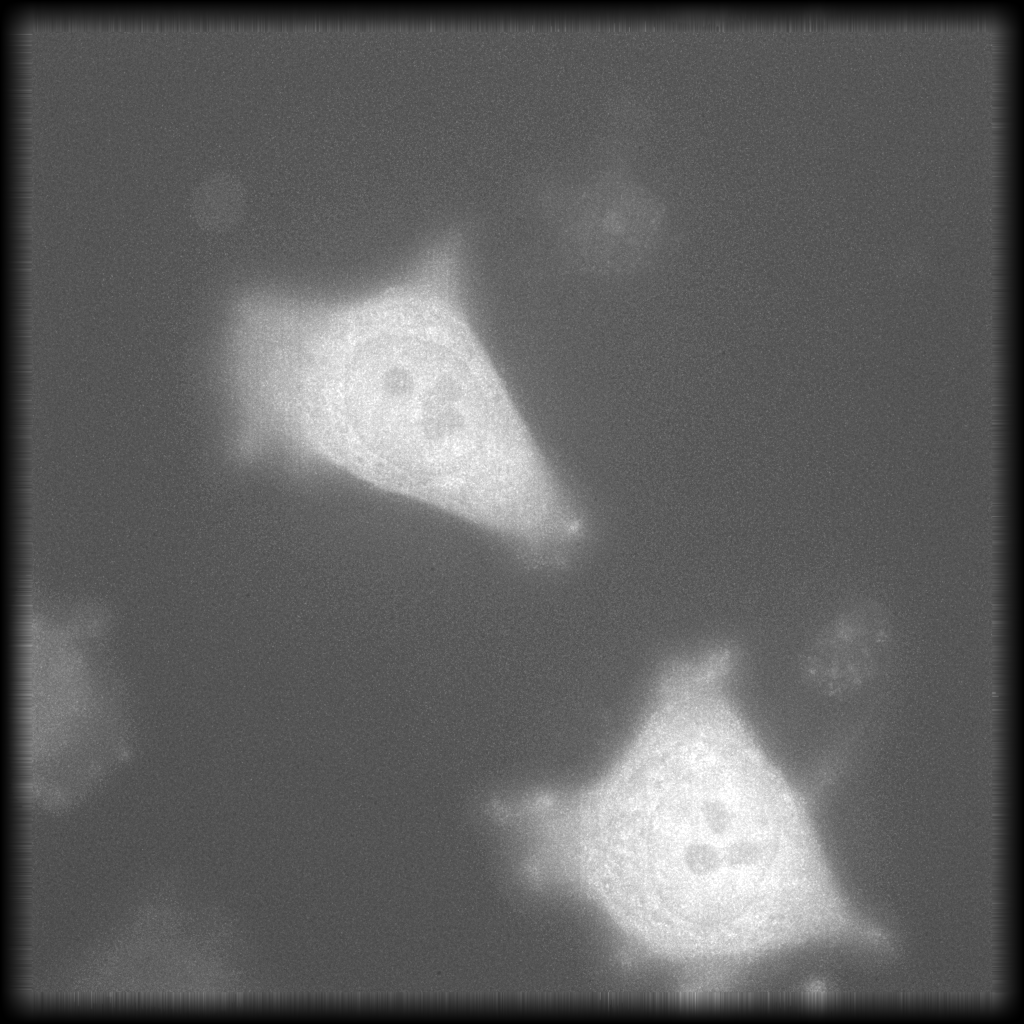

Supplement: Supplementary file 6 — Source data Fig. 1 [file 44319_2026_776_MOESM6_ESM.zip › Figure1/Figure1D_right.tif]

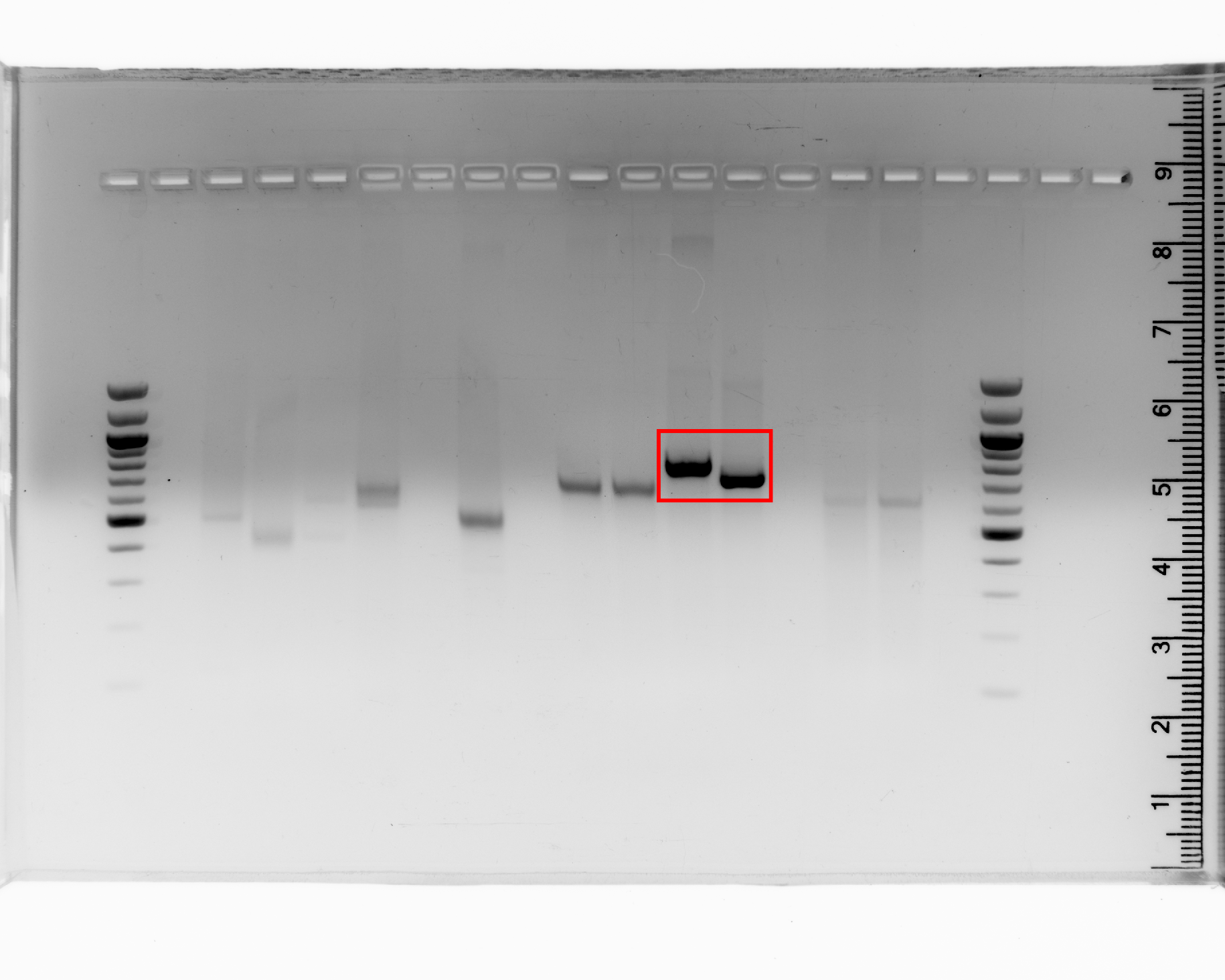

Supplement: Supplementary file 6 — Source data Fig. 1 [file 44319_2026_776_MOESM6_ESM.zip › Figure1/Figure1C_gel.tif]

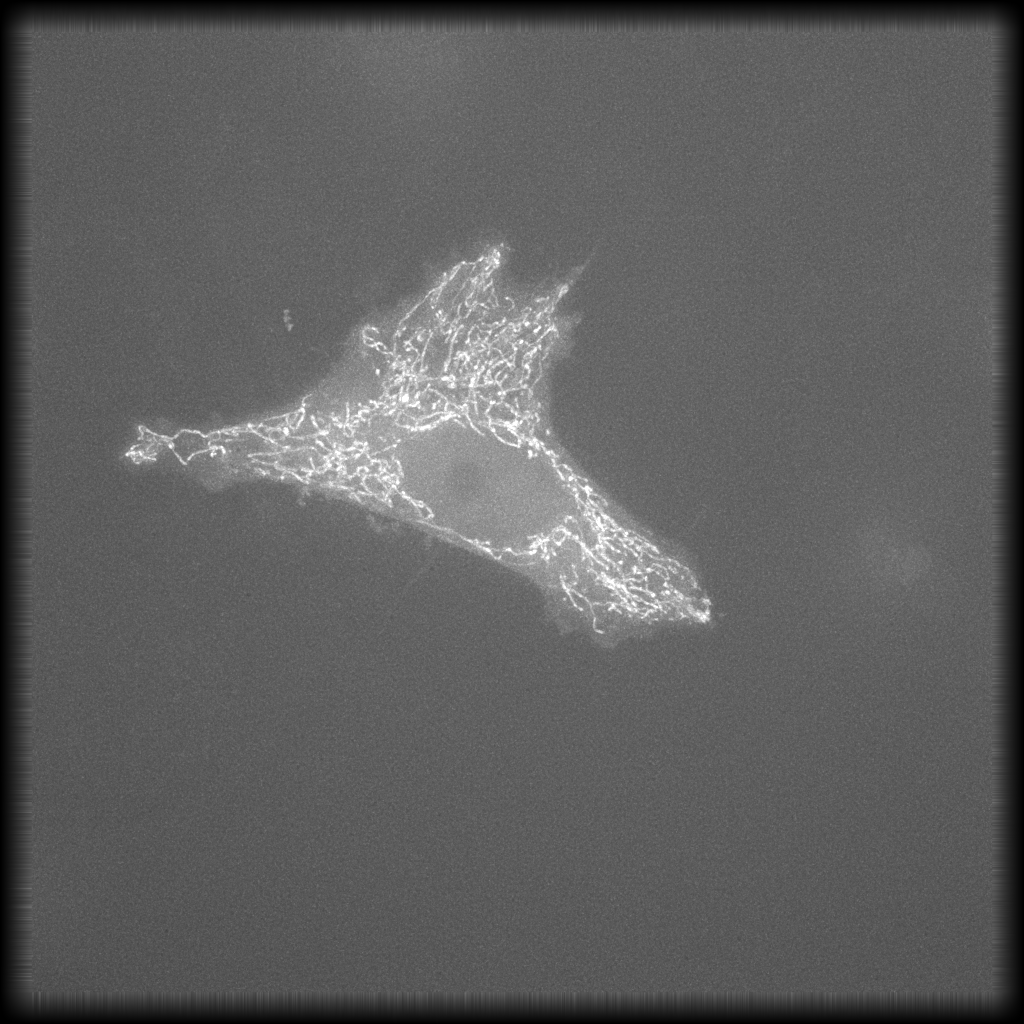

Supplement: Supplementary file 6 — Source data Fig. 1 [file 44319_2026_776_MOESM6_ESM.zip › Figure1/Figure1E_6nt.tif]

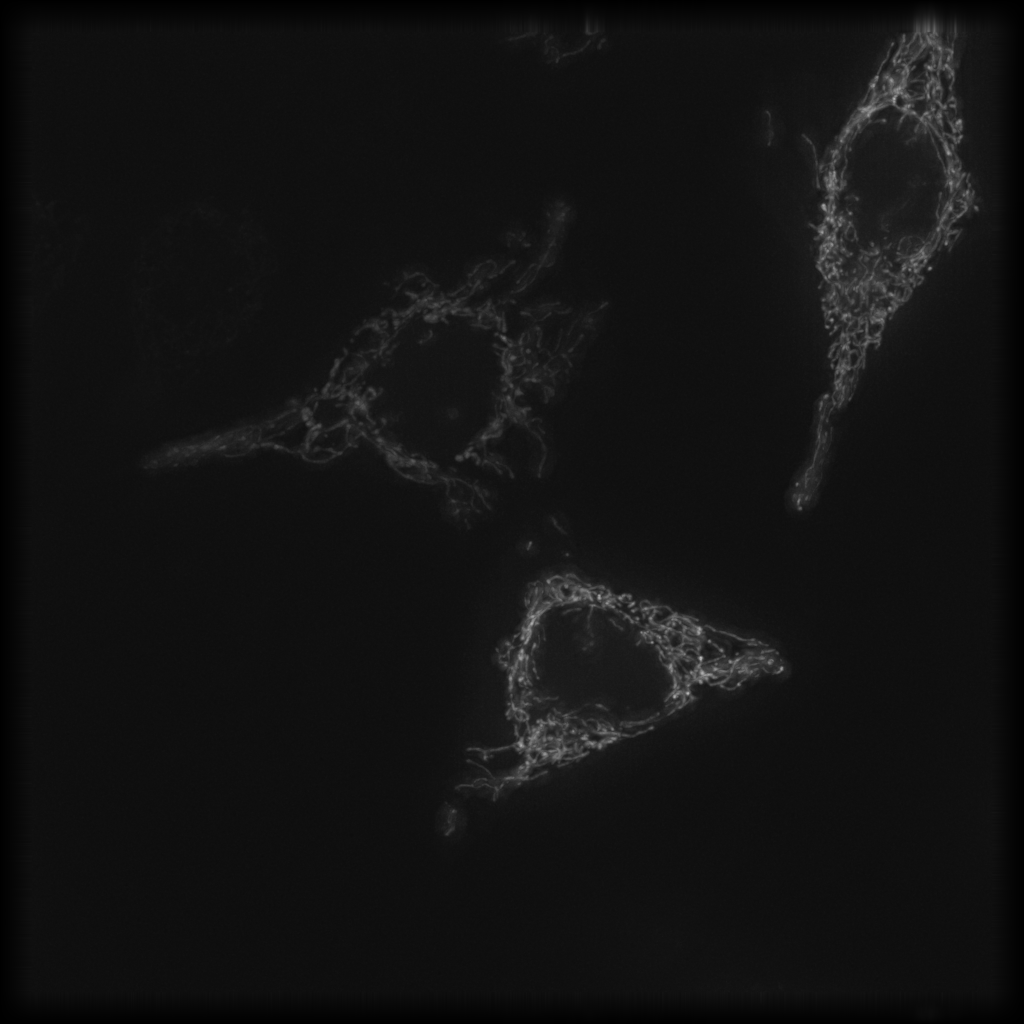

Supplement: Supplementary file 6 — Source data Fig. 1 [file 44319_2026_776_MOESM6_ESM.zip › Figure1/Figure1E_40nt.tif]

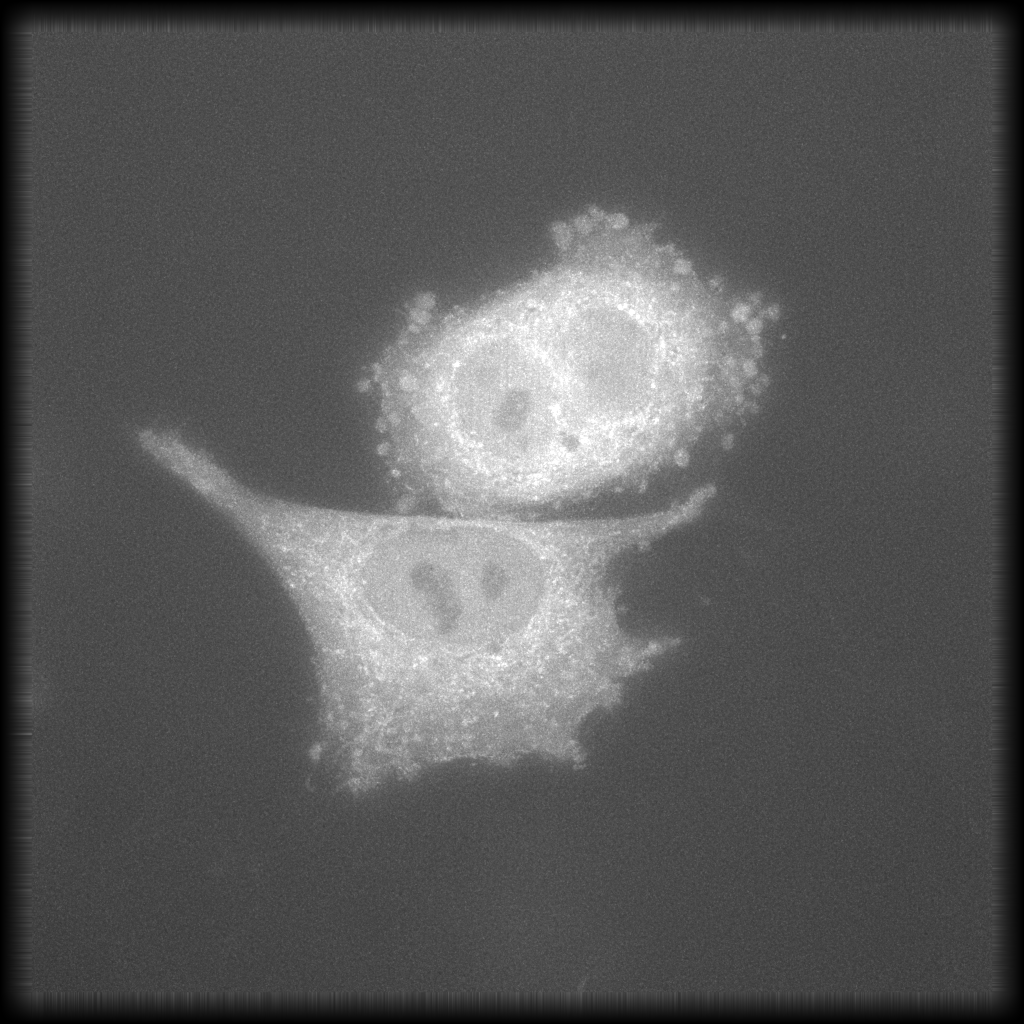

Supplement: Supplementary file 6 — Source data Fig. 1 [file 44319_2026_776_MOESM6_ESM.zip › Figure1/Figure1E_2nt.tif]

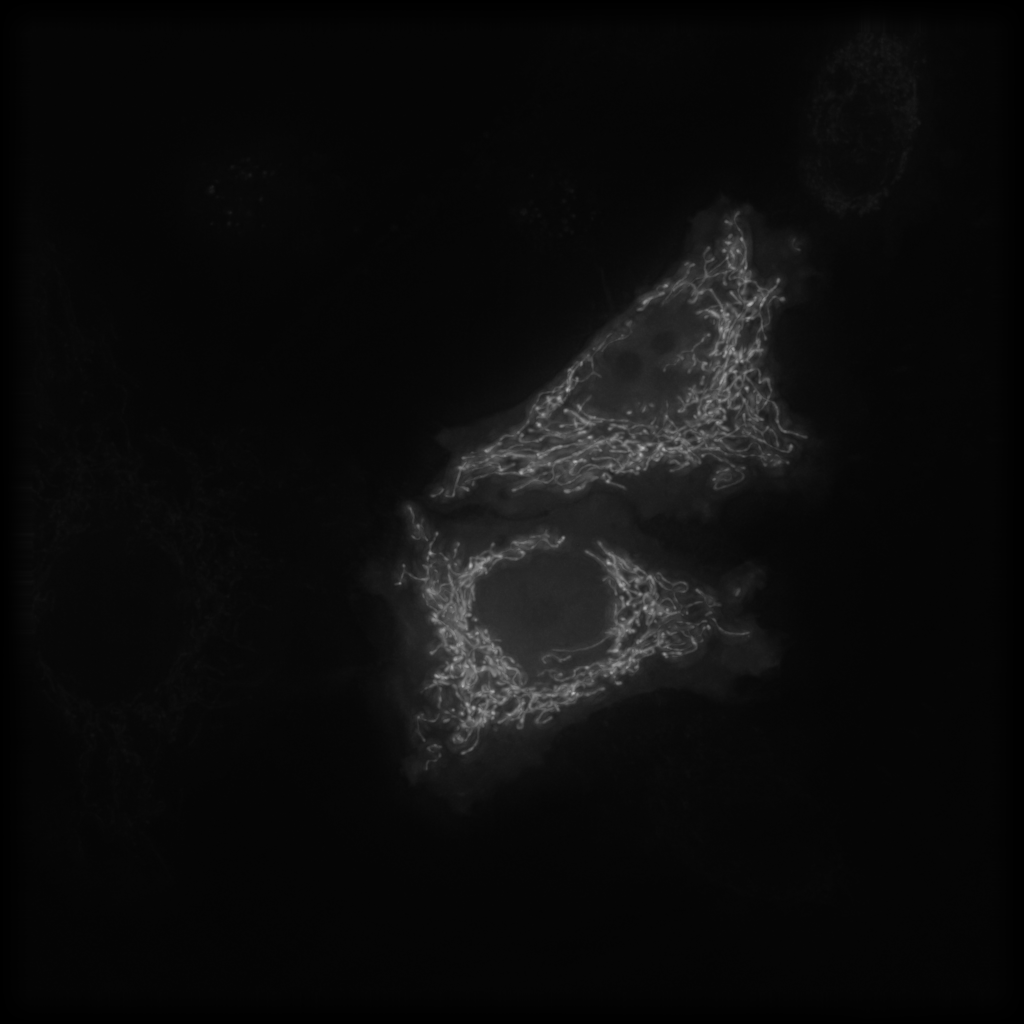

Supplement: Supplementary file 6 — Source data Fig. 1 [file 44319_2026_776_MOESM6_ESM.zip › Figure1/Figure1D_left.tif]

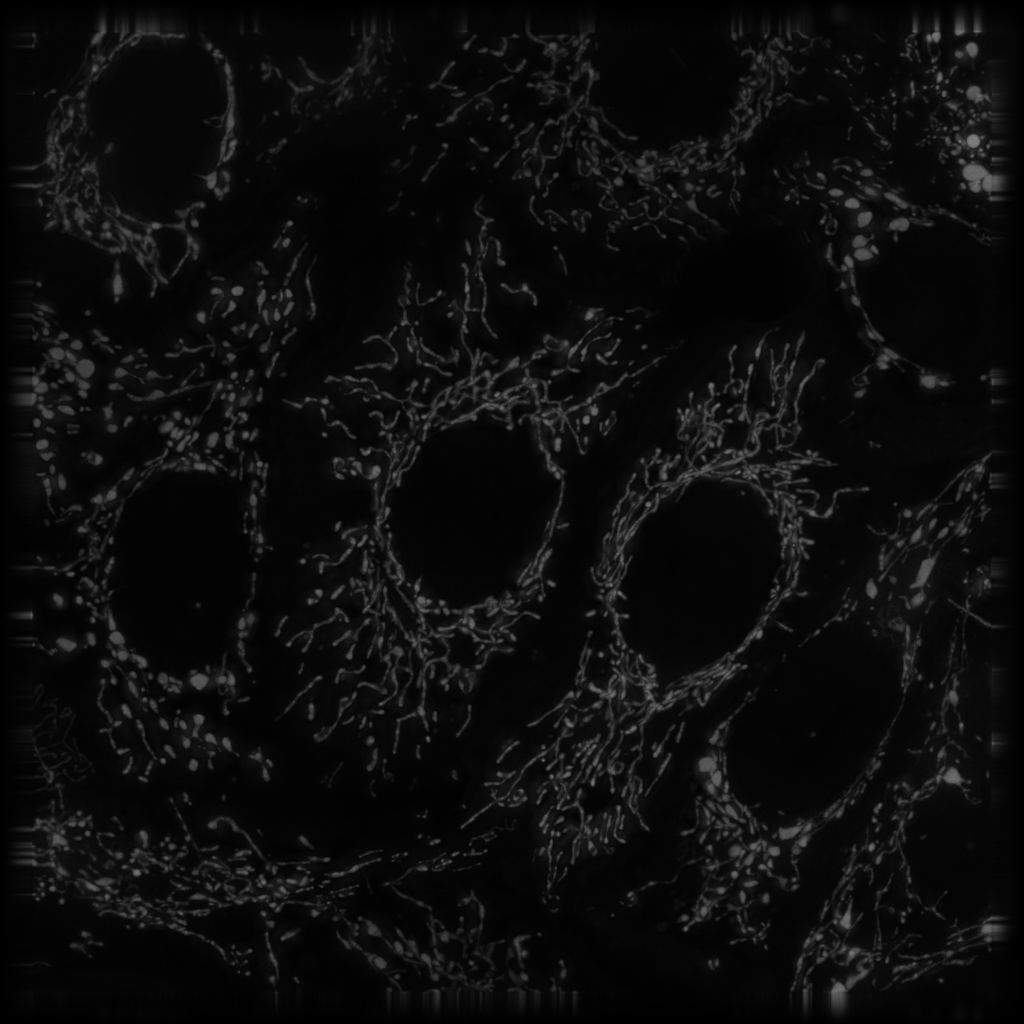

Supplement: Supplementary file 6 — Source data Fig. 1 [file 44319_2026_776_MOESM6_ESM.zip › Figure1/Figure1B_left.tif]

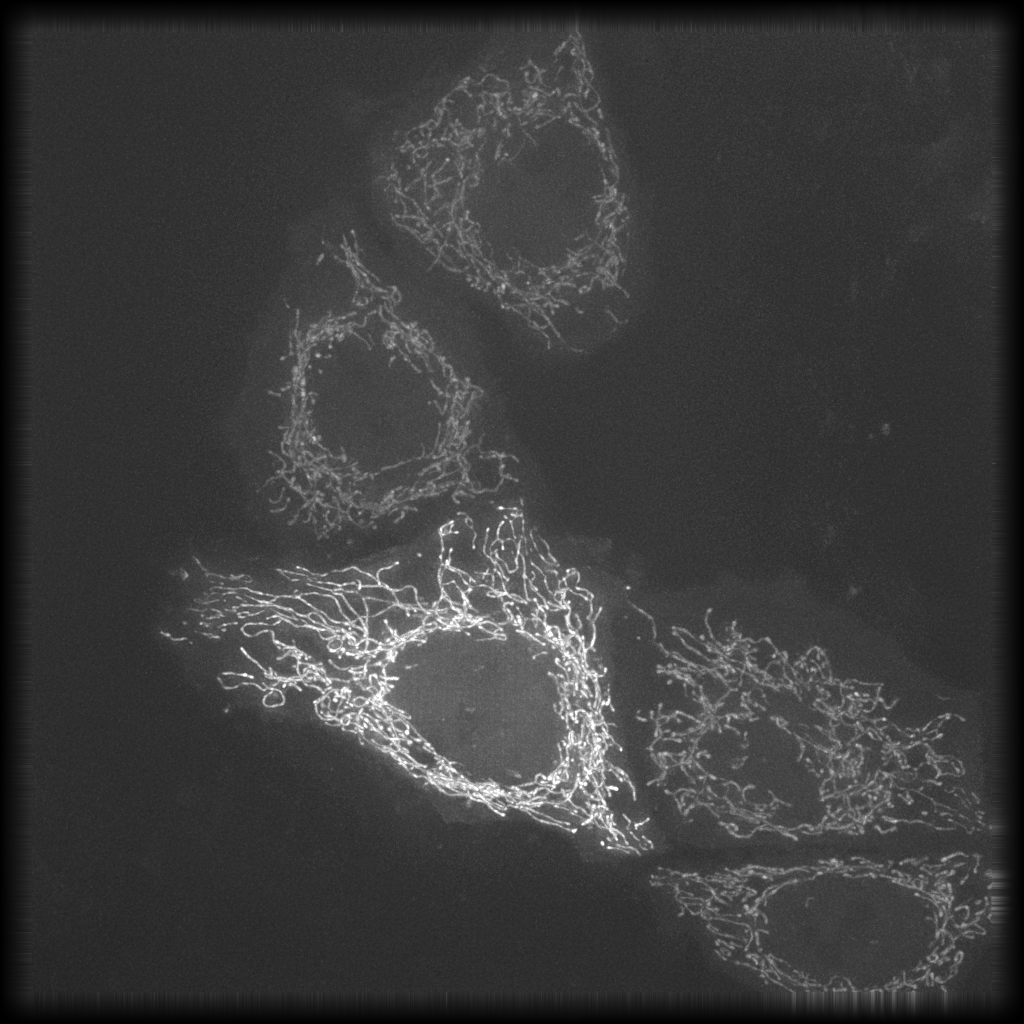

Supplement: Supplementary file 6 — Source data Fig. 1 [file 44319_2026_776_MOESM6_ESM.zip › Figure1/Figure1E_20nt.tif]

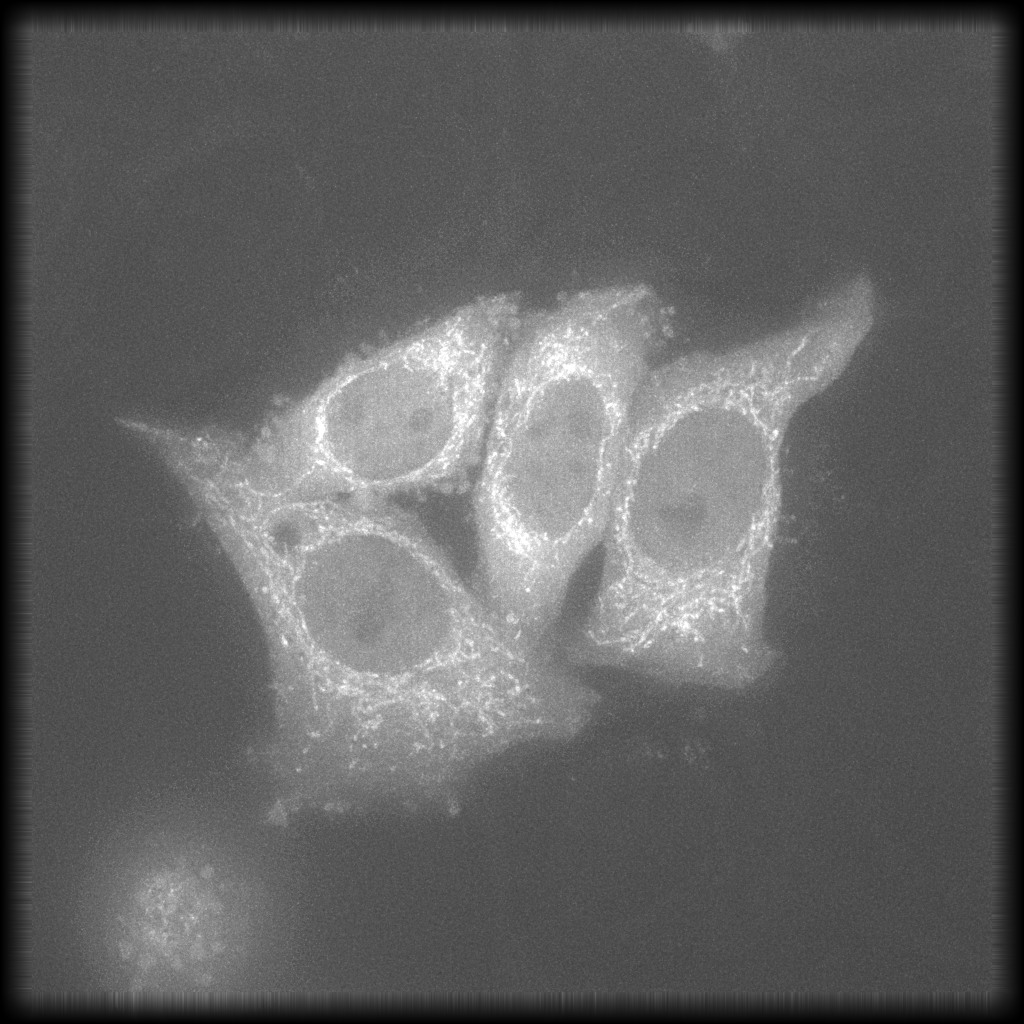

Supplement: Supplementary file 7 — Source data Fig. 2 [file 44319_2026_776_MOESM7_ESM.zip › Figure2/Figure2D_left.tif]

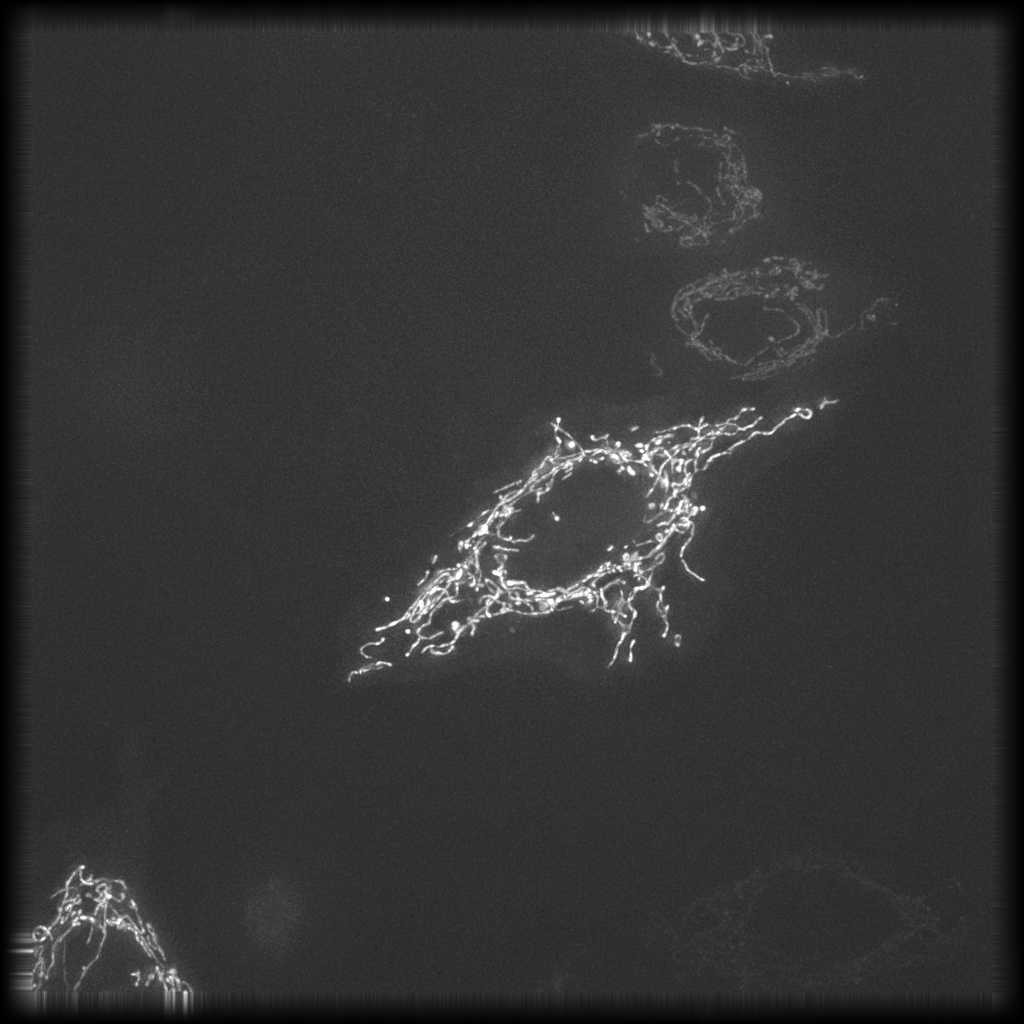

Supplement: Supplementary file 7 — Source data Fig. 2 [file 44319_2026_776_MOESM7_ESM.zip › Figure2/Figure2D_right.tif]

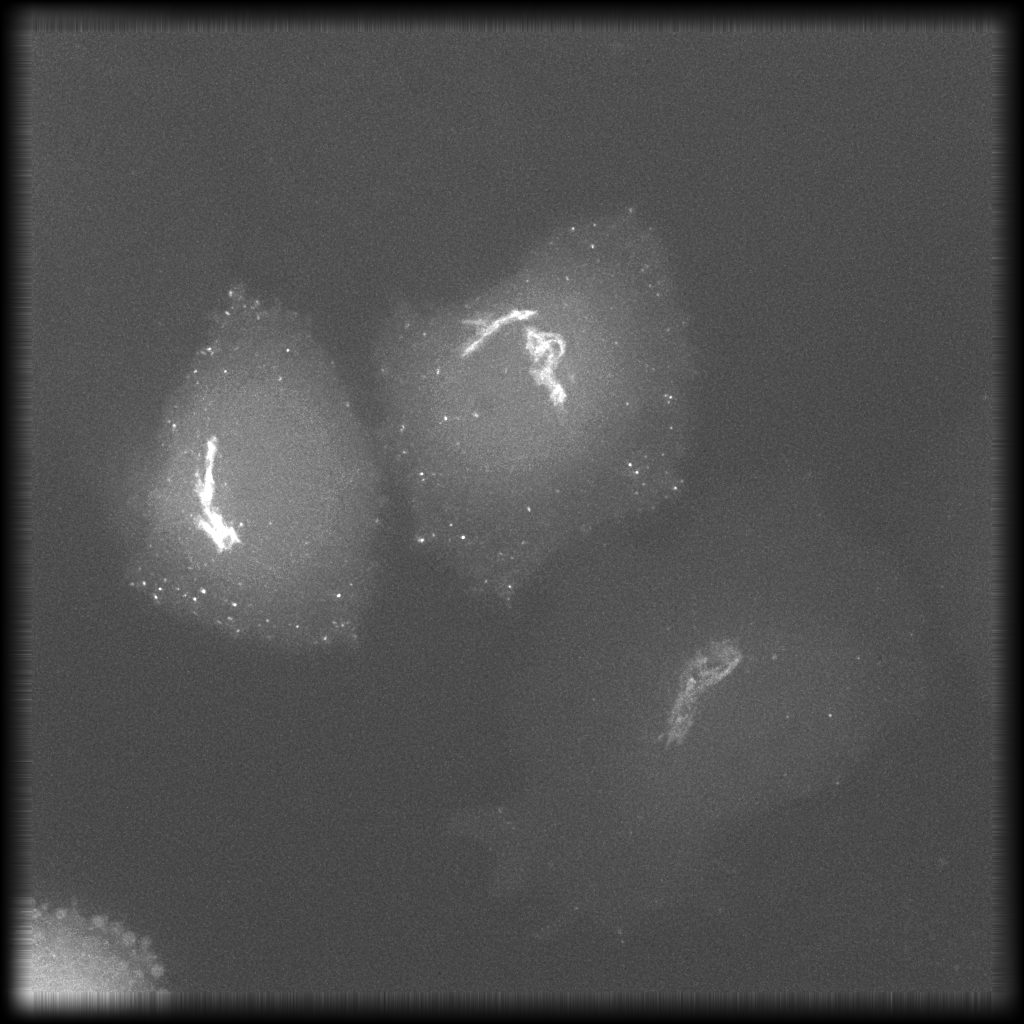

Supplement: Supplementary file 8 — Source data Fig. 3 [file 44319_2026_776_MOESM8_ESM.zip › Figure3/Figure3C_right.tif]

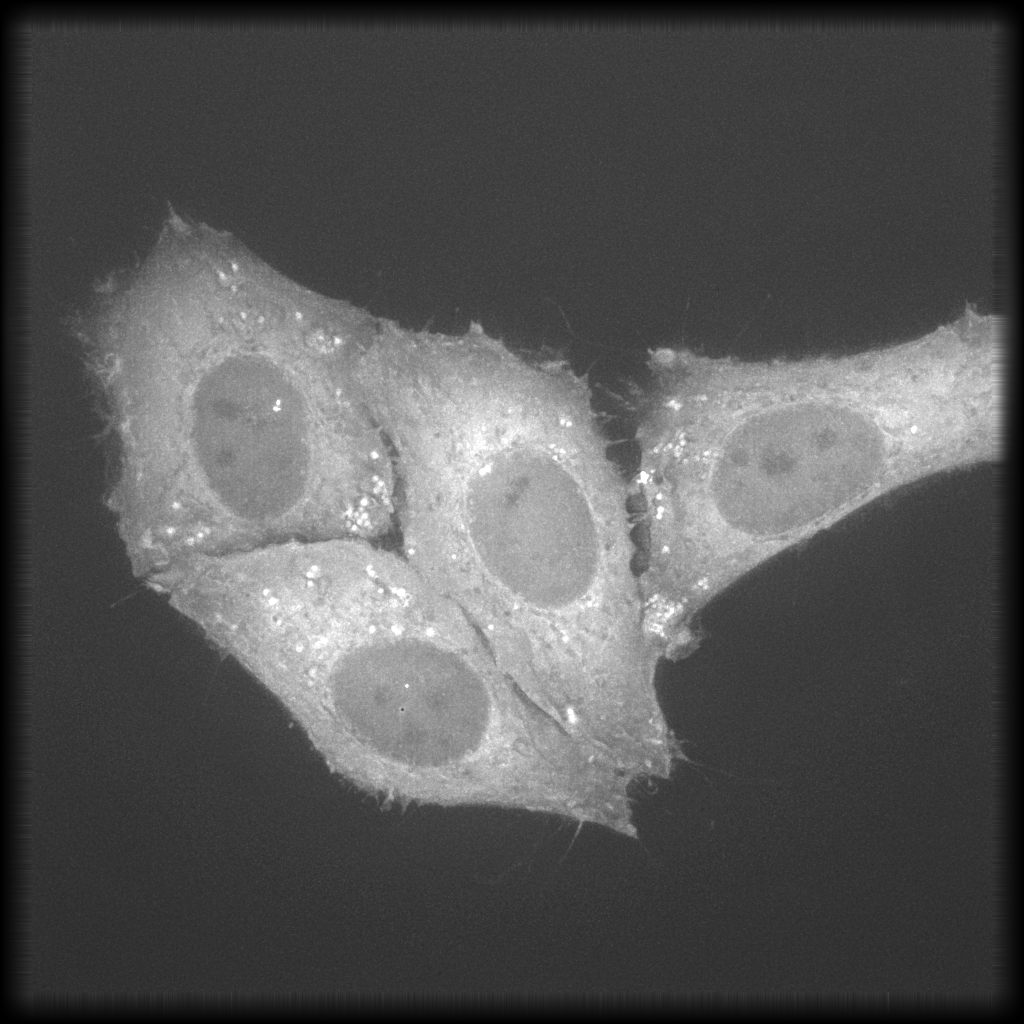

Supplement: Supplementary file 8 — Source data Fig. 3 [file 44319_2026_776_MOESM8_ESM.zip › Figure3/Figure3C_middle.tif]

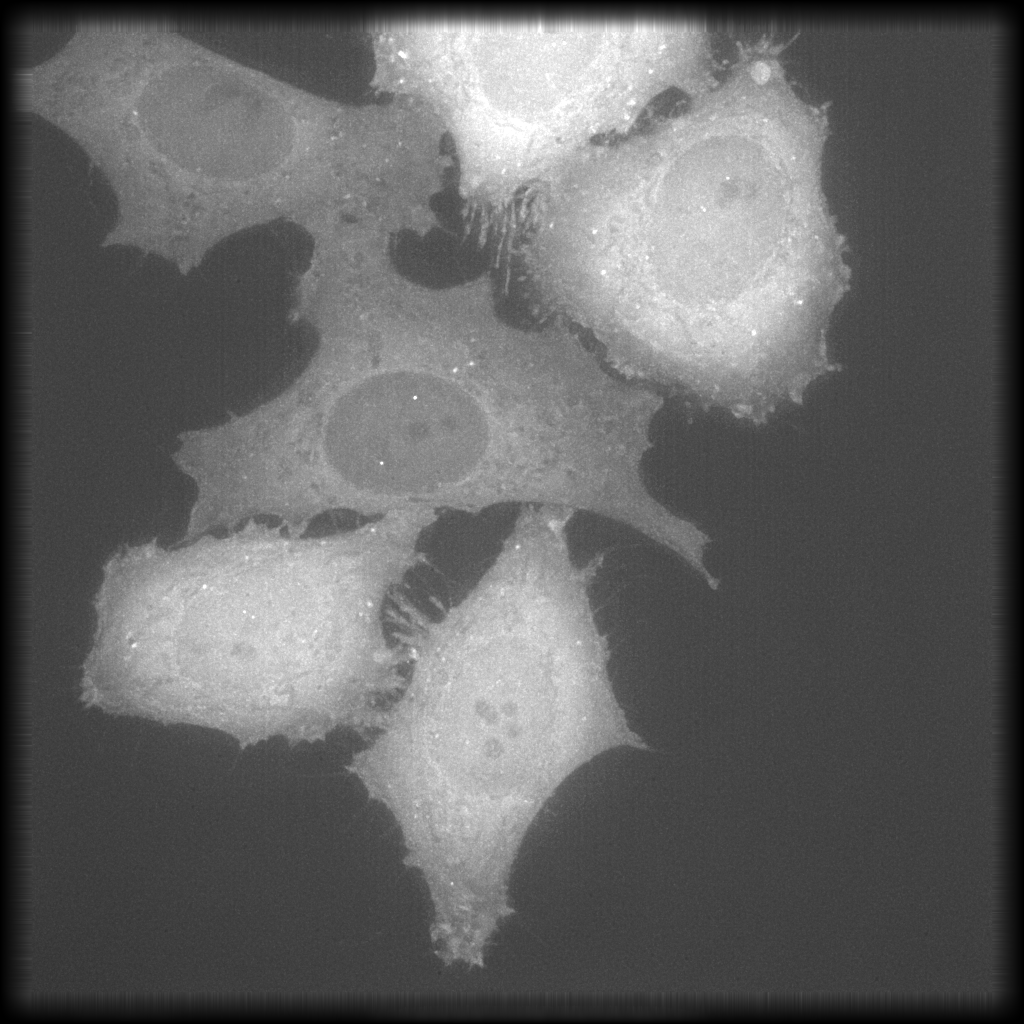

Supplement: Supplementary file 8 — Source data Fig. 3 [file 44319_2026_776_MOESM8_ESM.zip › Figure3/Figure3E_right.tif]

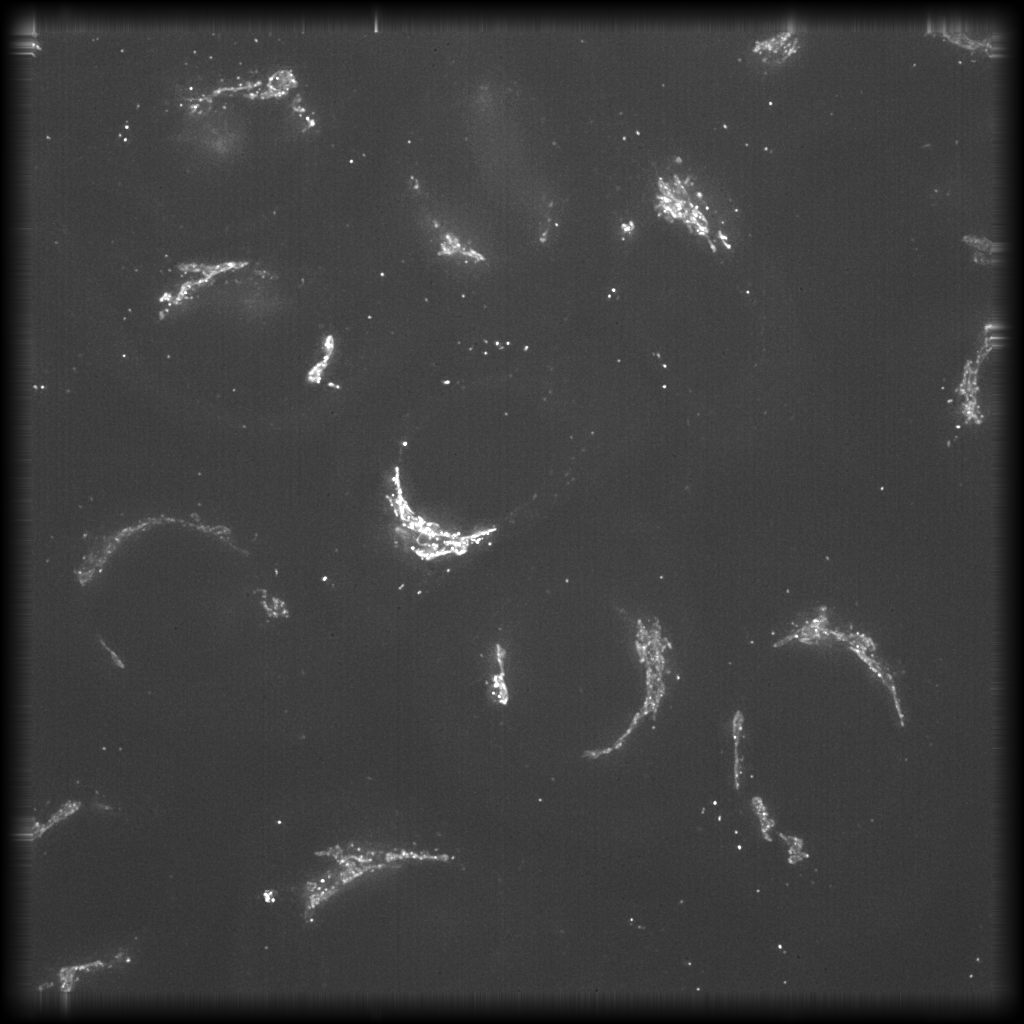

Supplement: Supplementary file 8 — Source data Fig. 3 [file 44319_2026_776_MOESM8_ESM.zip › Figure3/Figure3E_left.tif]

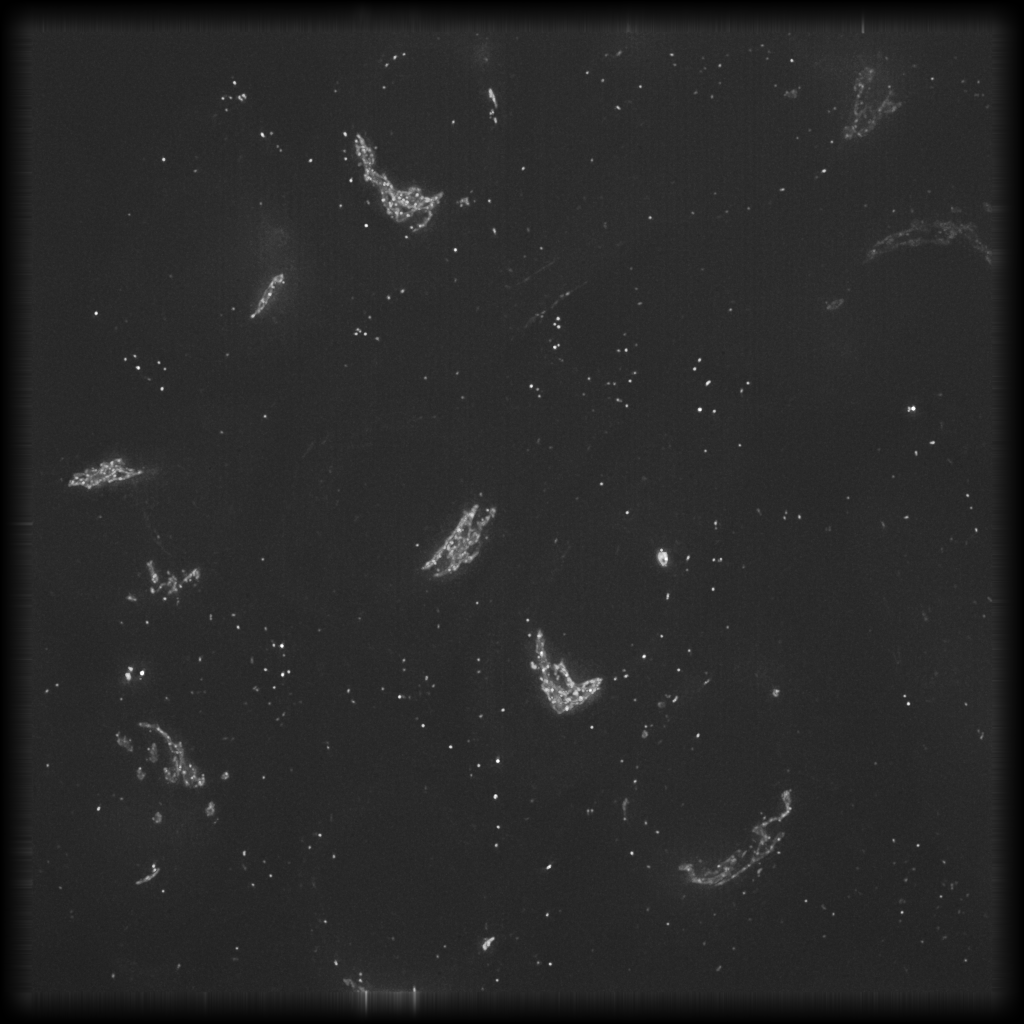

Supplement: Supplementary file 8 — Source data Fig. 3 [file 44319_2026_776_MOESM8_ESM.zip › Figure3/Figure3C_left.tif]

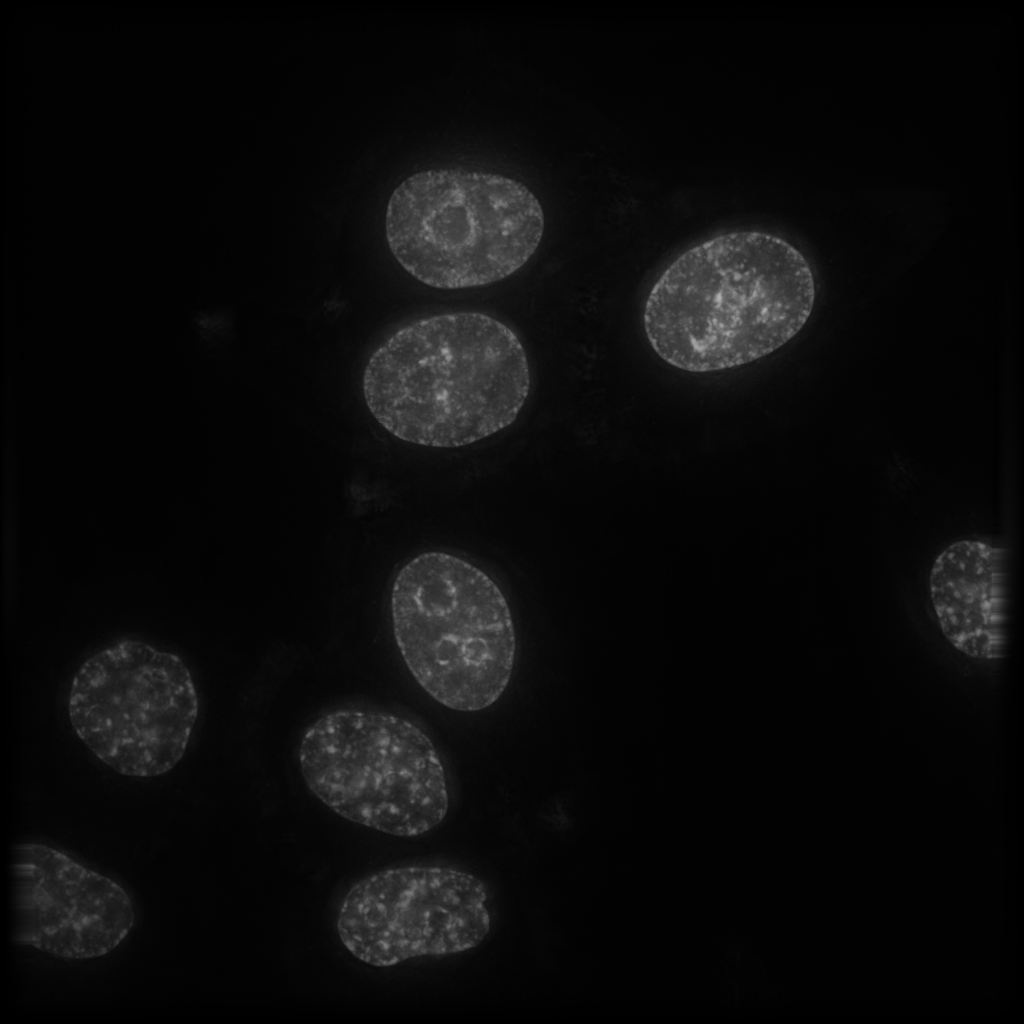

Supplement: Supplementary file 9 — Source data Fig. 4 [file 44319_2026_776_MOESM9_ESM.zip › Figure4/Figure4C_left.tif]

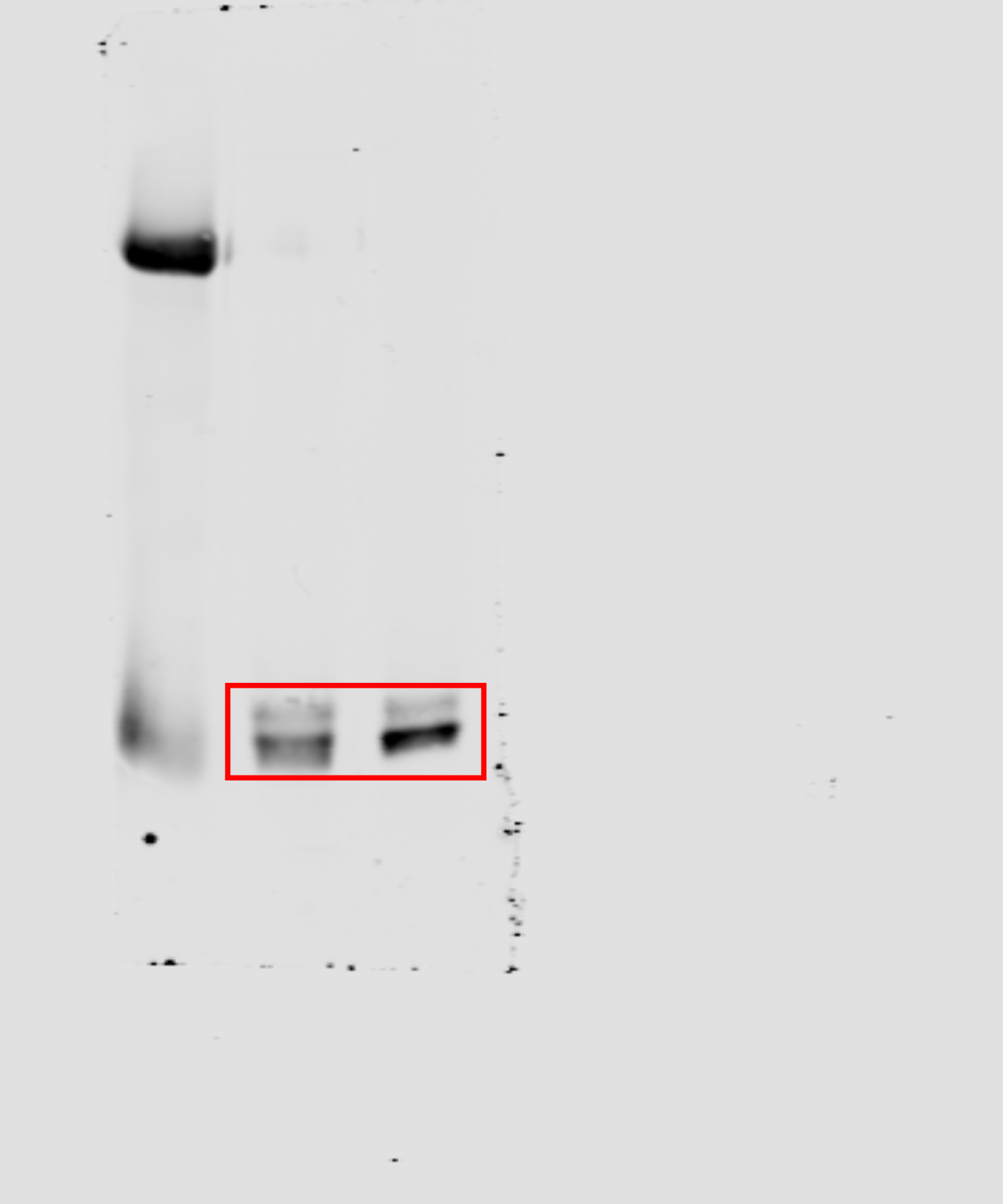

Supplement: Supplementary file 9 — Source data Fig. 4 [file 44319_2026_776_MOESM9_ESM.zip › Figure4/Figure4F_GFPblot.tif]

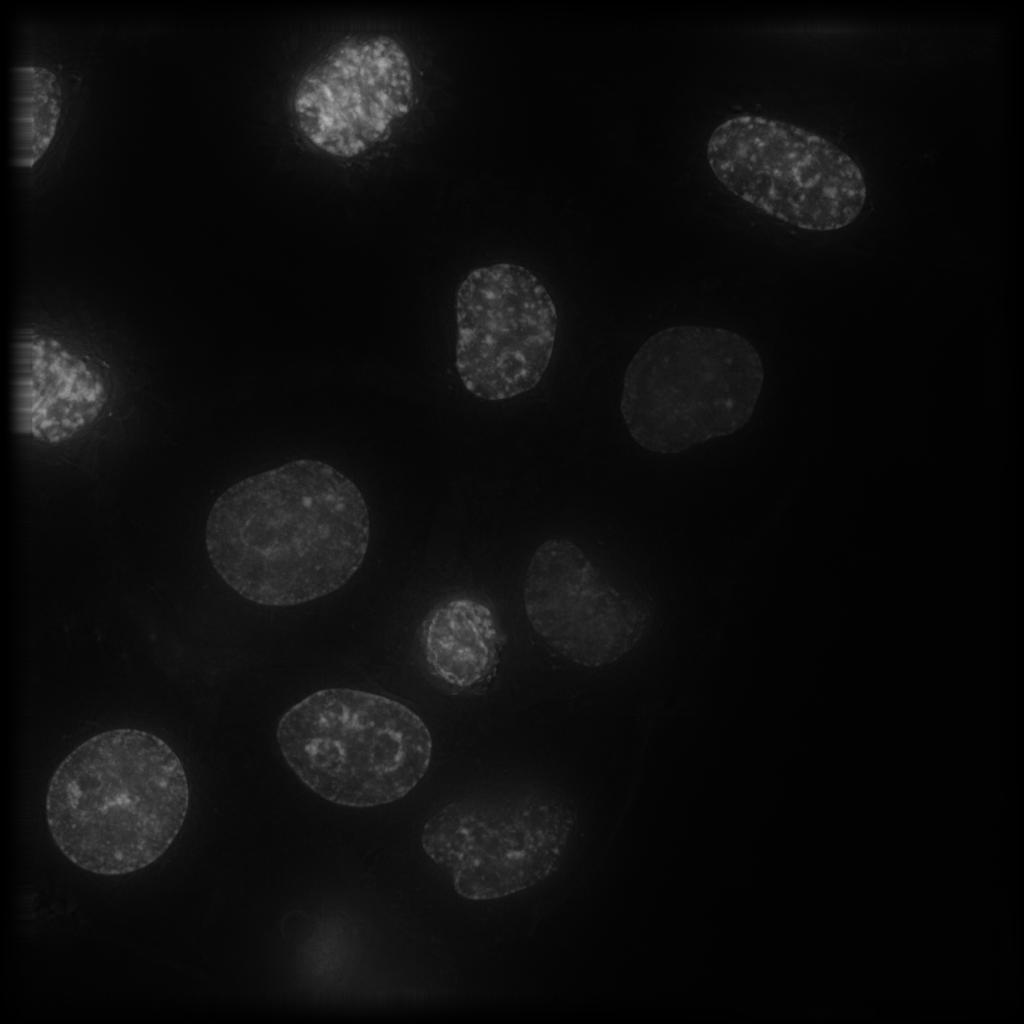

Supplement: Supplementary file 9 — Source data Fig. 4 [file 44319_2026_776_MOESM9_ESM.zip › Figure4/Figure4C_right.tif]

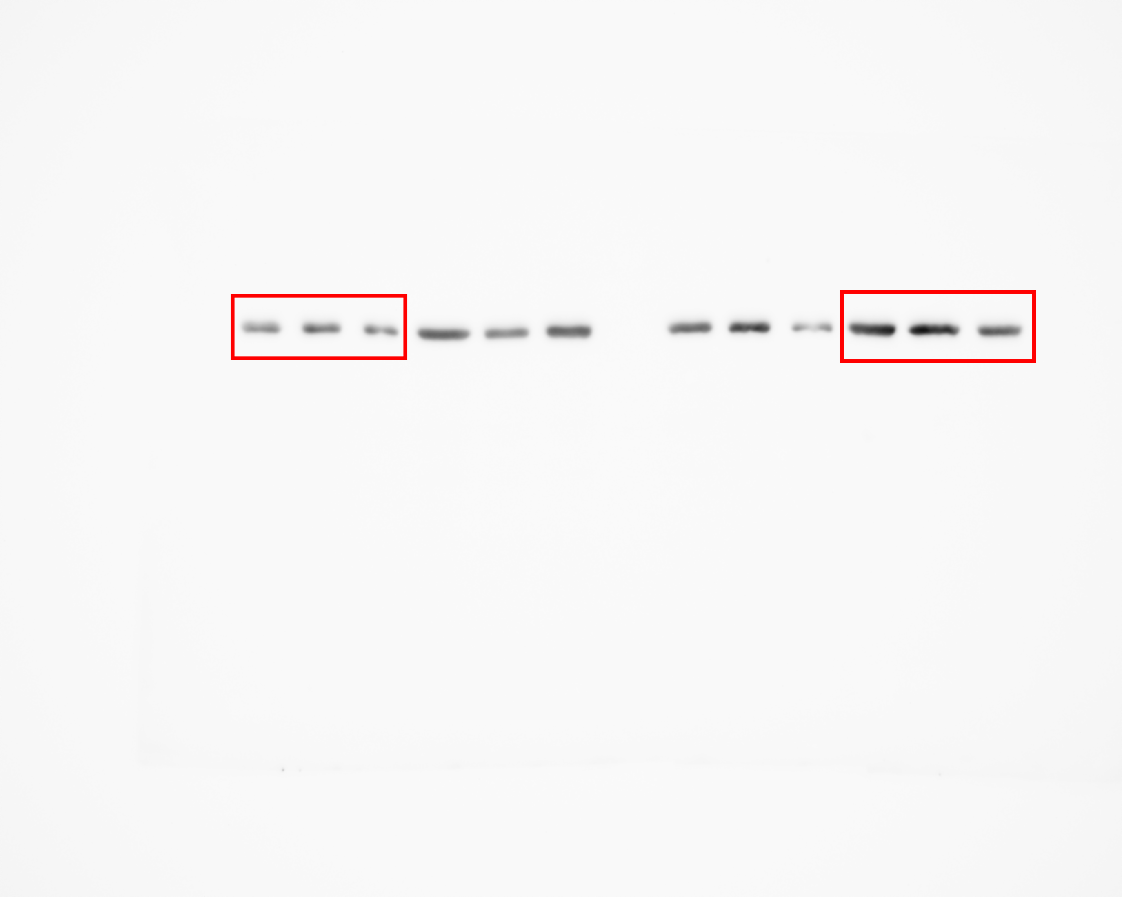

Supplement: Supplementary file 10 — Source data Fig. 5 [file 44319_2026_776_MOESM10_ESM.zip › Figure5/Figure5D_tubulin_blot.tif]

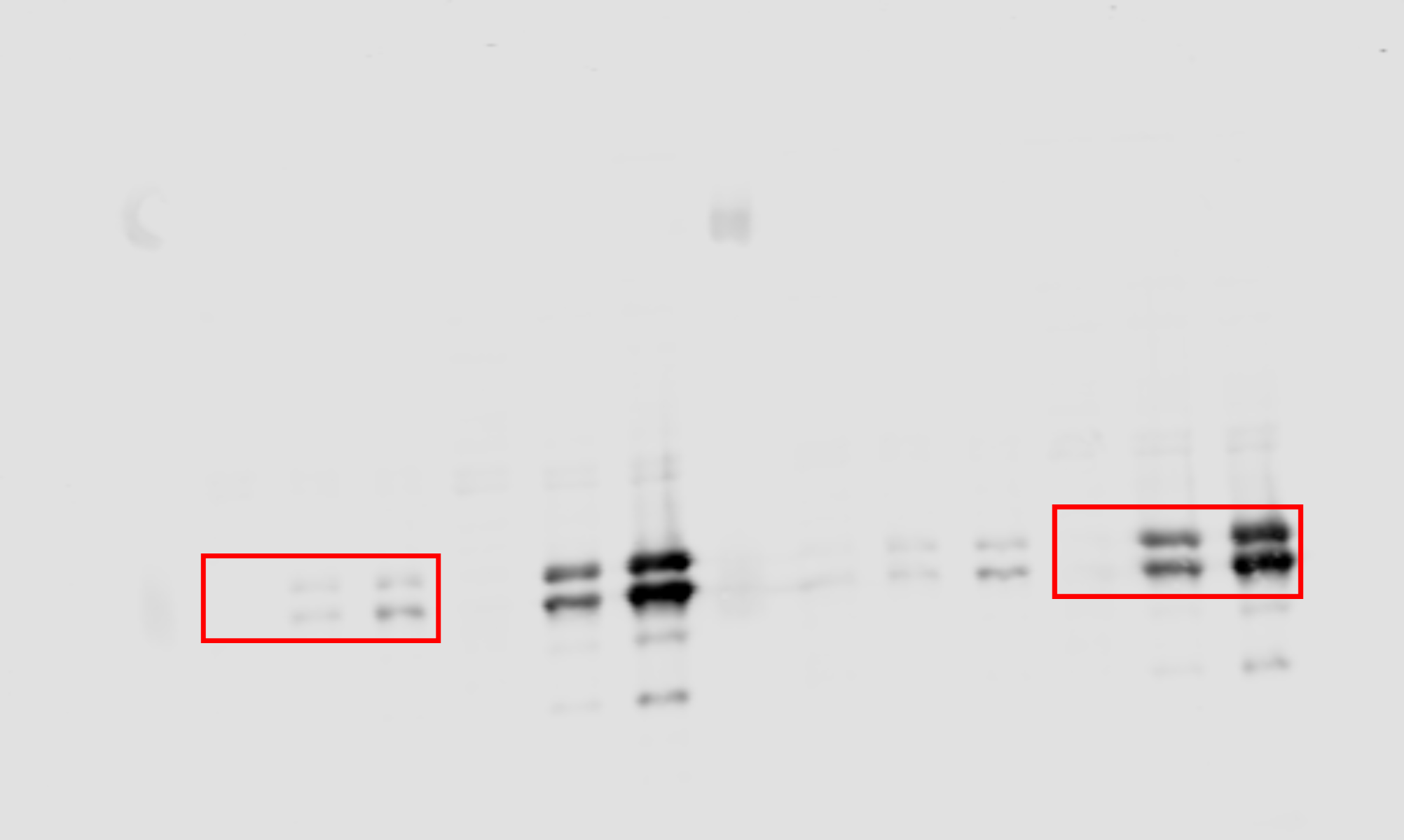

Supplement: Supplementary file 10 — Source data Fig. 5 [file 44319_2026_776_MOESM10_ESM.zip › Figure5/Figure5D_GFP_blot.tif]

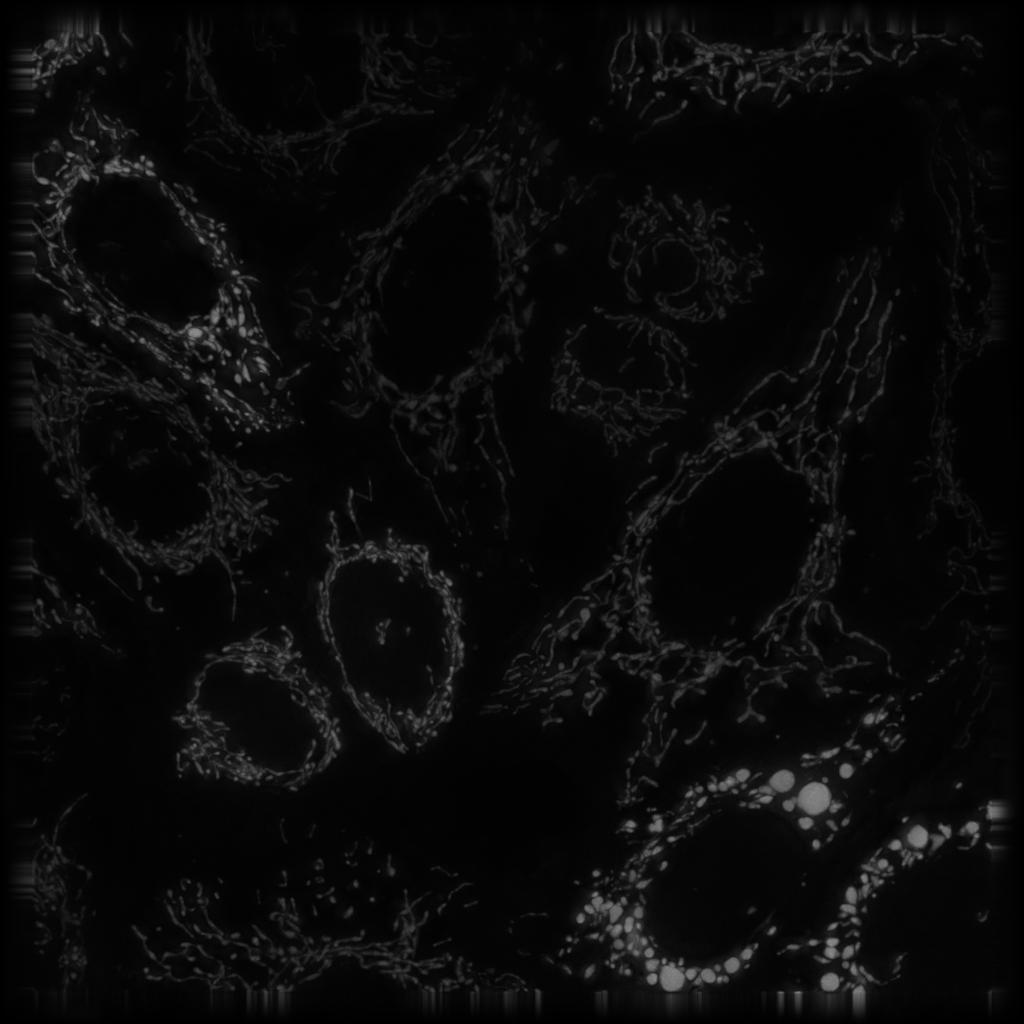

Supplement: Supplementary file 11 — Figure EV1 Source Data [file 44319_2026_776_MOESM11_ESM.zip › FigureEV1/FigureEV1A_left.tif]

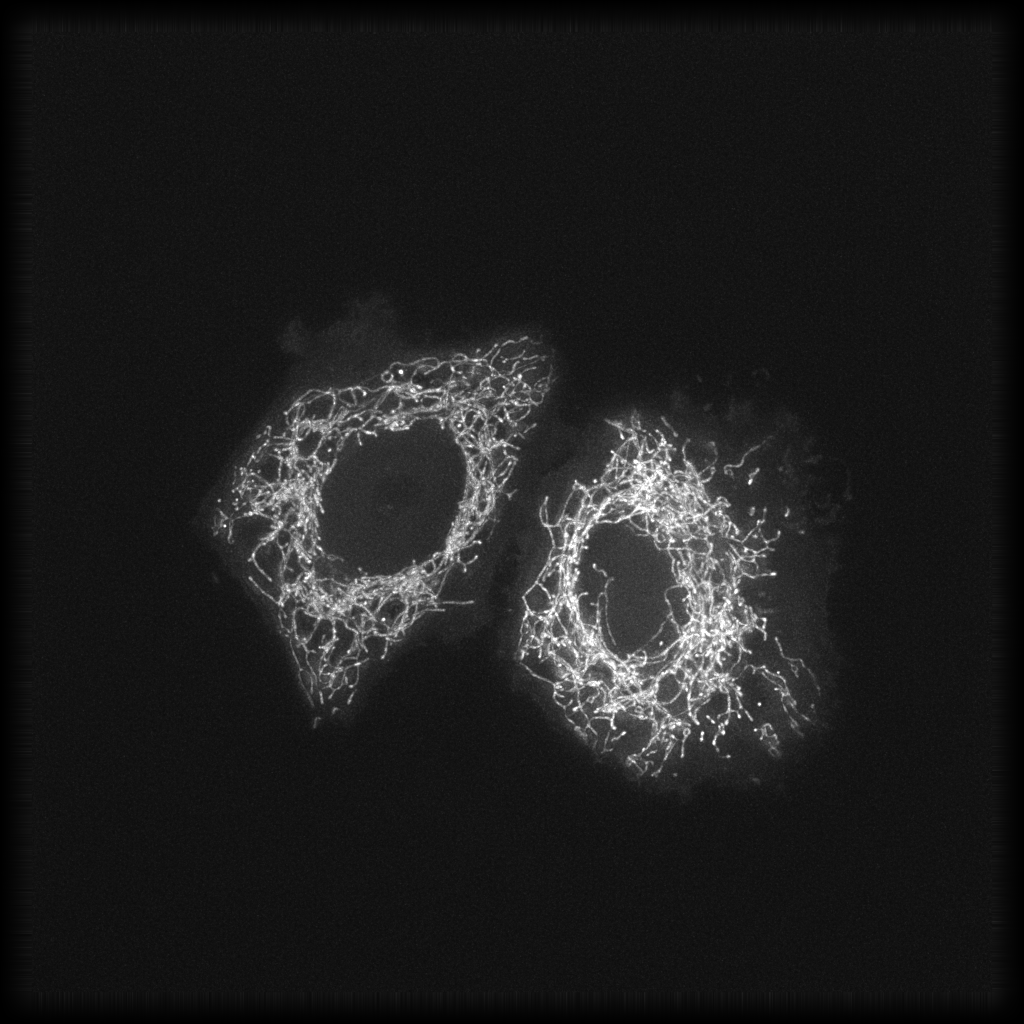

Supplement: Supplementary file 11 — Figure EV1 Source Data [file 44319_2026_776_MOESM11_ESM.zip › FigureEV1/FigureEV1C_right.tif]

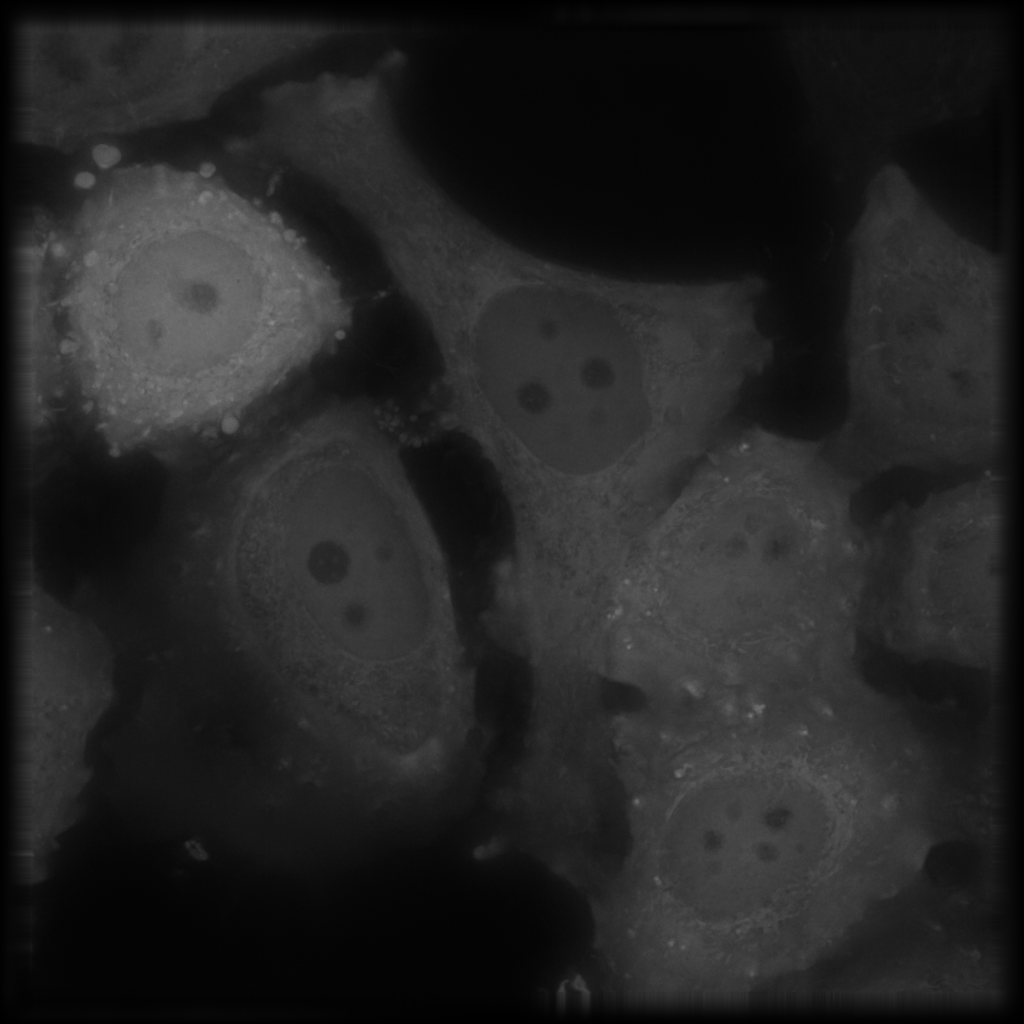

Supplement: Supplementary file 11 — Figure EV1 Source Data [file 44319_2026_776_MOESM11_ESM.zip › FigureEV1/FigureEV1A_right.tif]

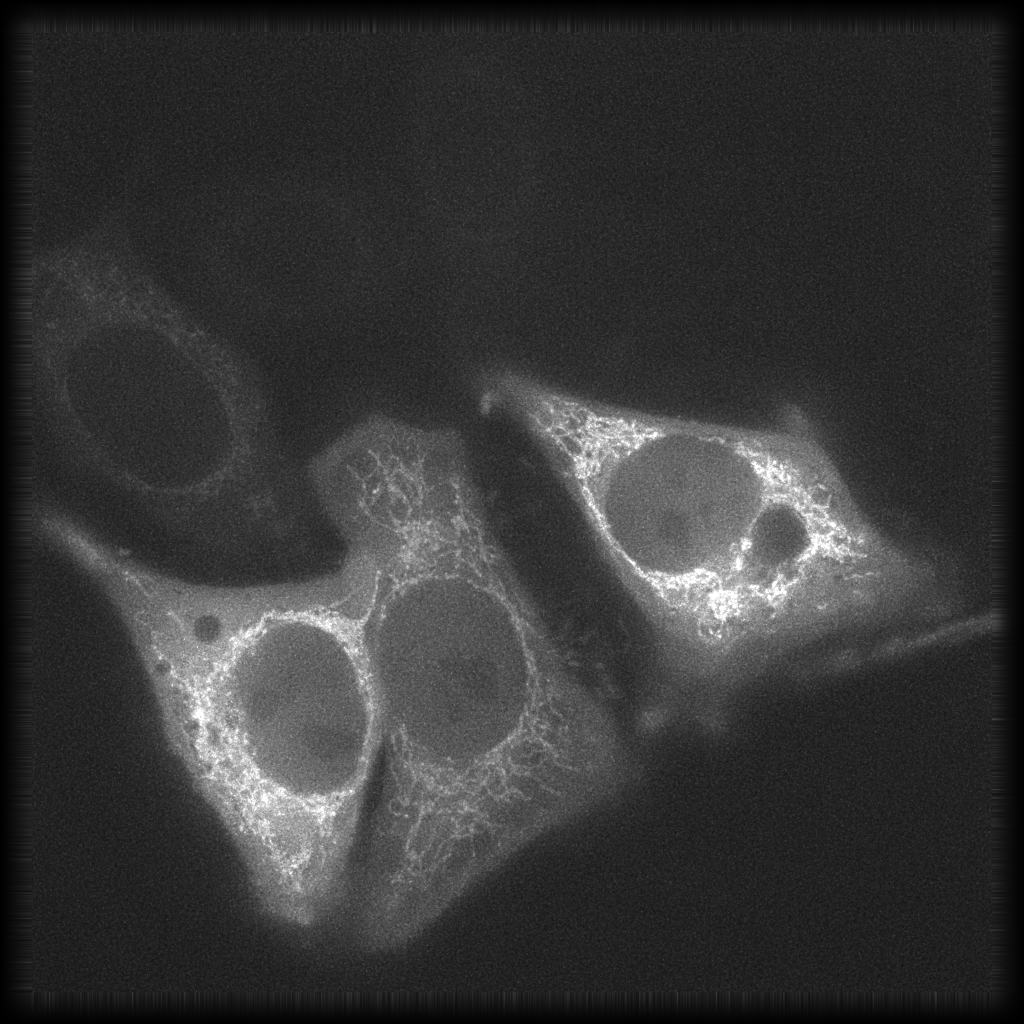

Supplement: Supplementary file 11 — Figure EV1 Source Data [file 44319_2026_776_MOESM11_ESM.zip › FigureEV1/FigureEV1C_left.tif]

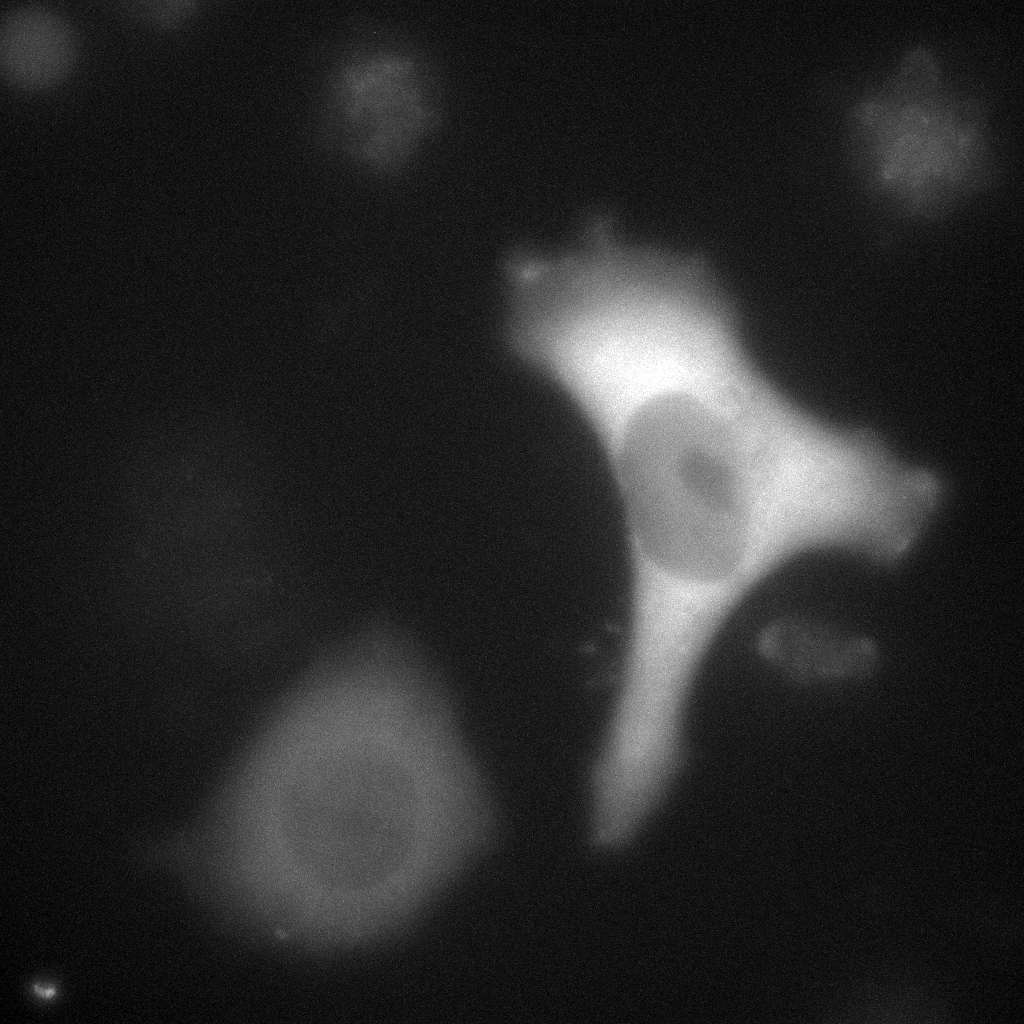

Supplement: Supplementary file 13 — Figure EV3 Source Data [file 44319_2026_776_MOESM13_ESM.zip › FigureEV3/FigureEV3B_right.tif]

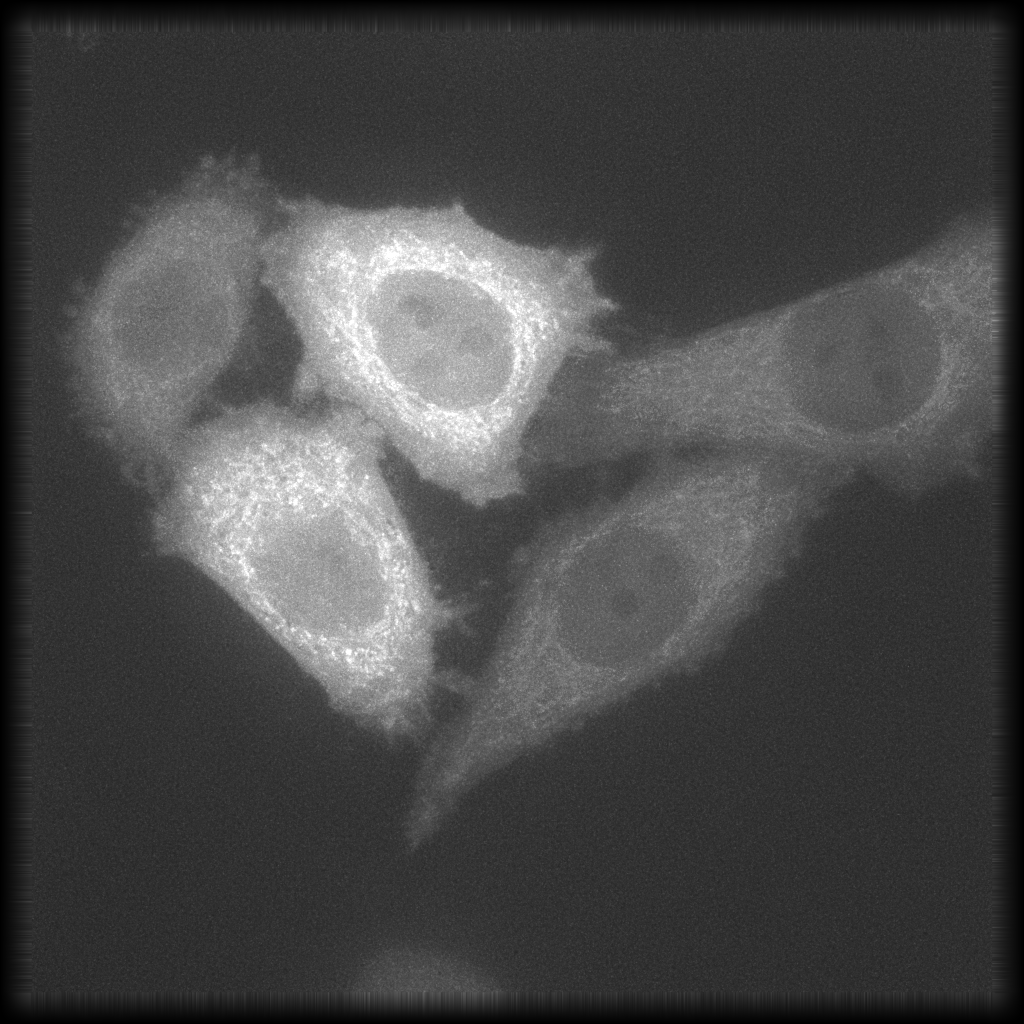

Supplement: Supplementary file 13 — Figure EV3 Source Data [file 44319_2026_776_MOESM13_ESM.zip › FigureEV3/FigureEV3B_left.tif]

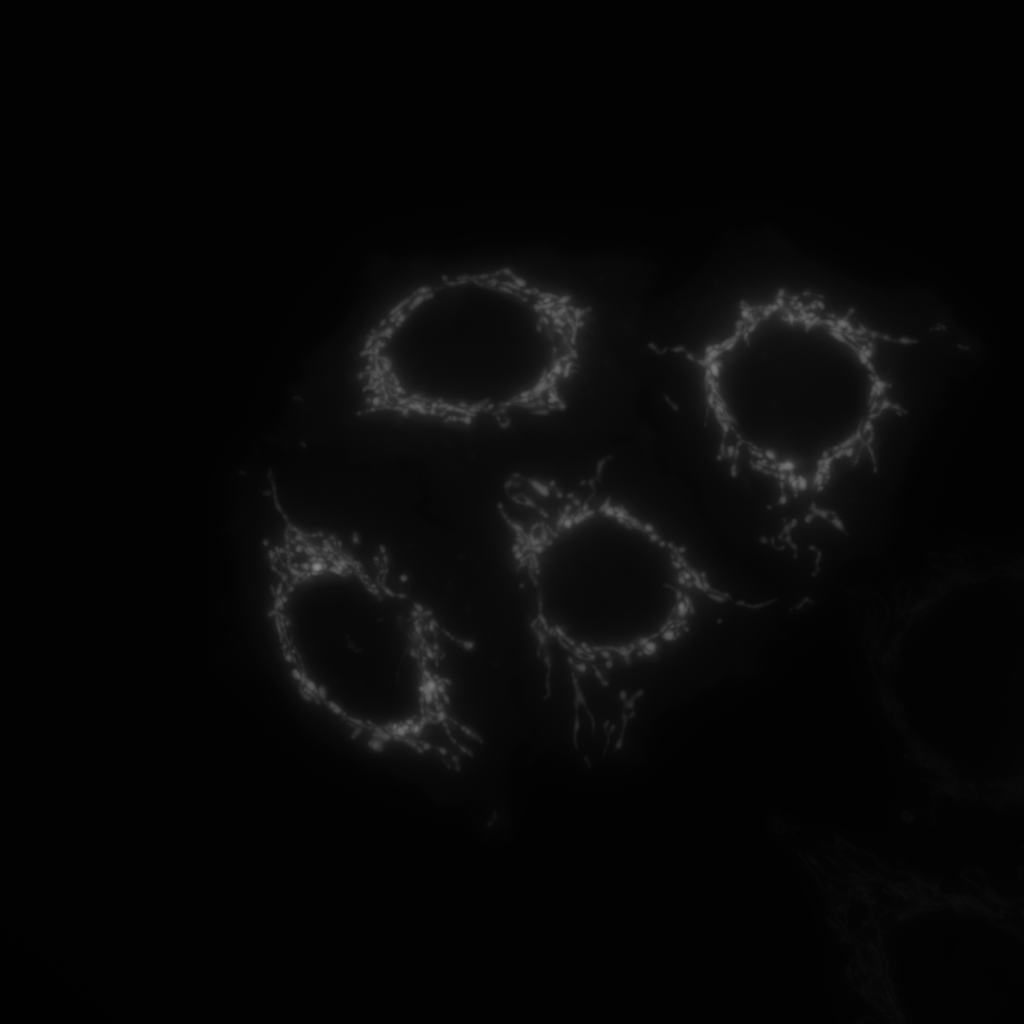

Supplement: Supplementary file 13 — Figure EV3 Source Data [file 44319_2026_776_MOESM13_ESM.zip › FigureEV3/FigureEV3A_left.tif]

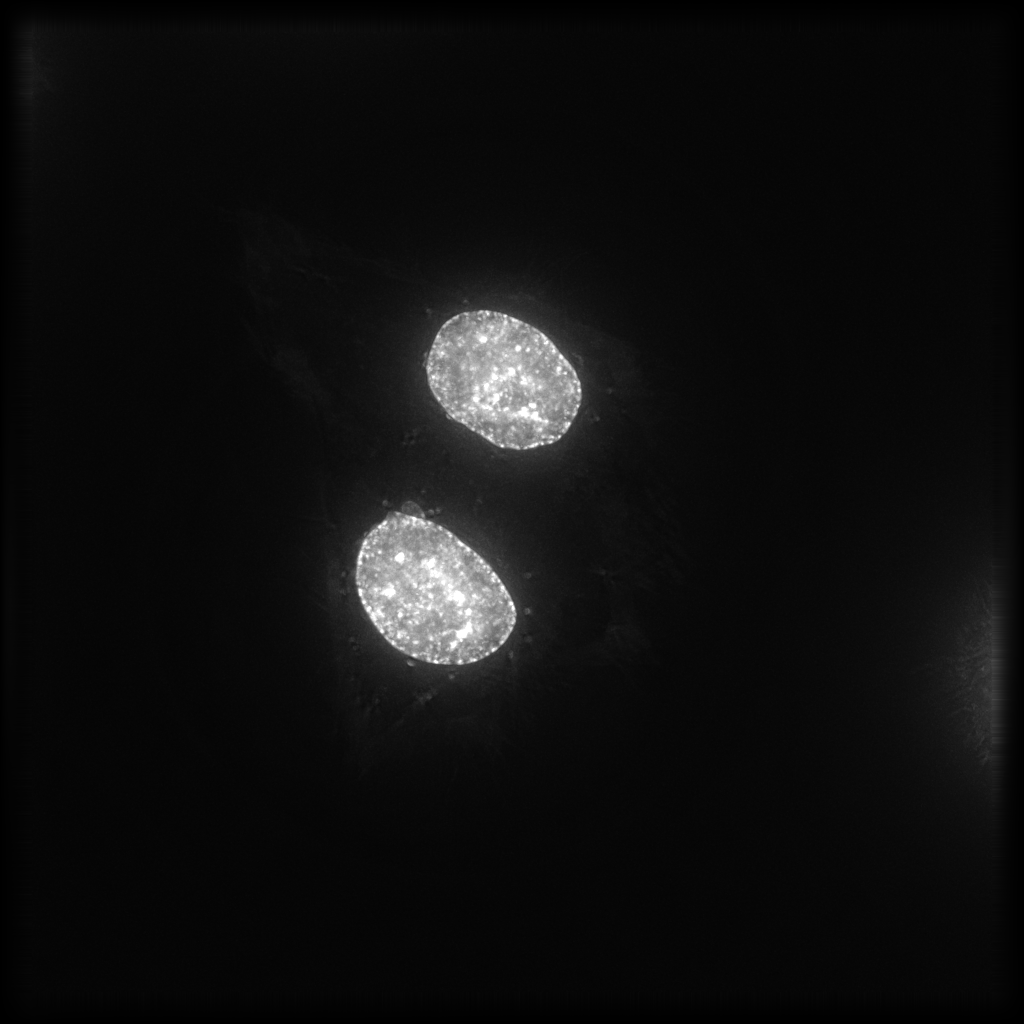

Supplement: Supplementary file 13 — Figure EV3 Source Data [file 44319_2026_776_MOESM13_ESM.zip › FigureEV3/FigureEV3F.tif]

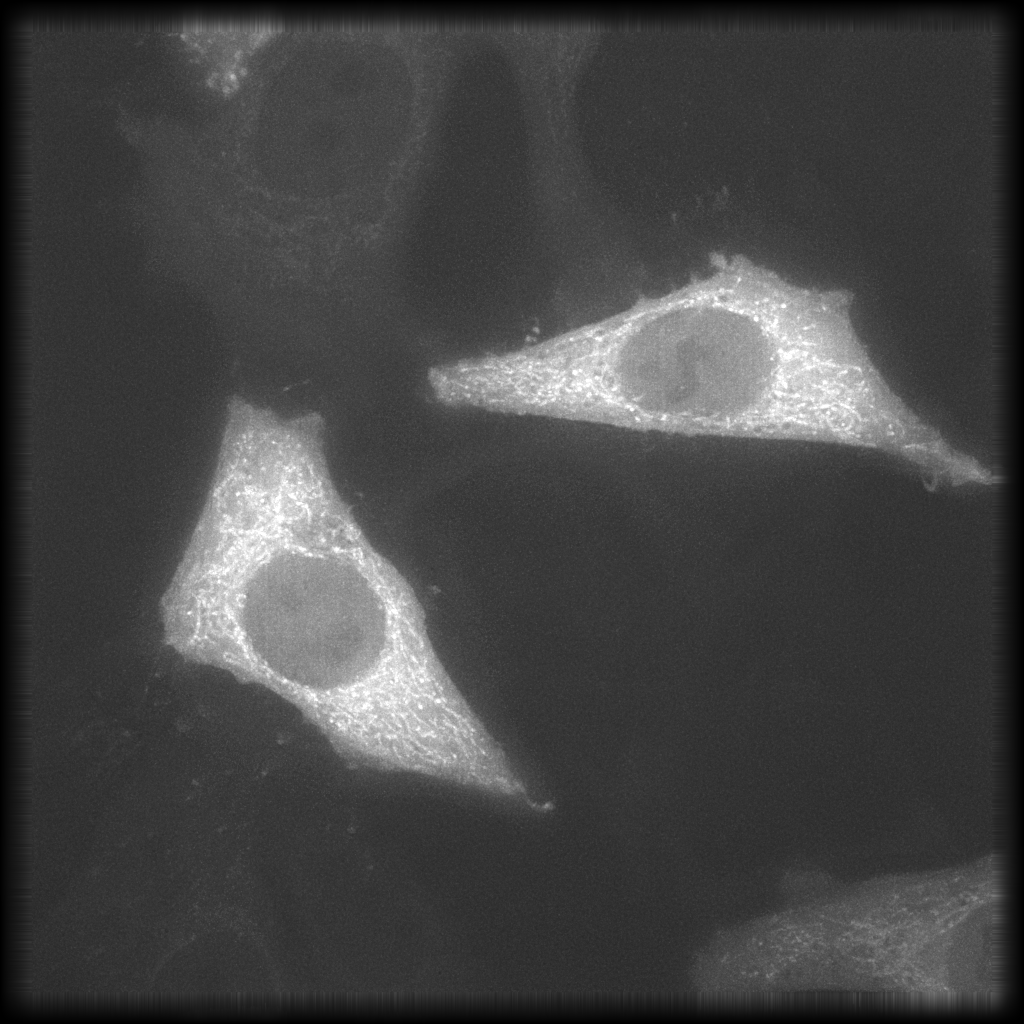

Supplement: Supplementary file 13 — Figure EV3 Source Data [file 44319_2026_776_MOESM13_ESM.zip › FigureEV3/FigureEV3A_right.tif]

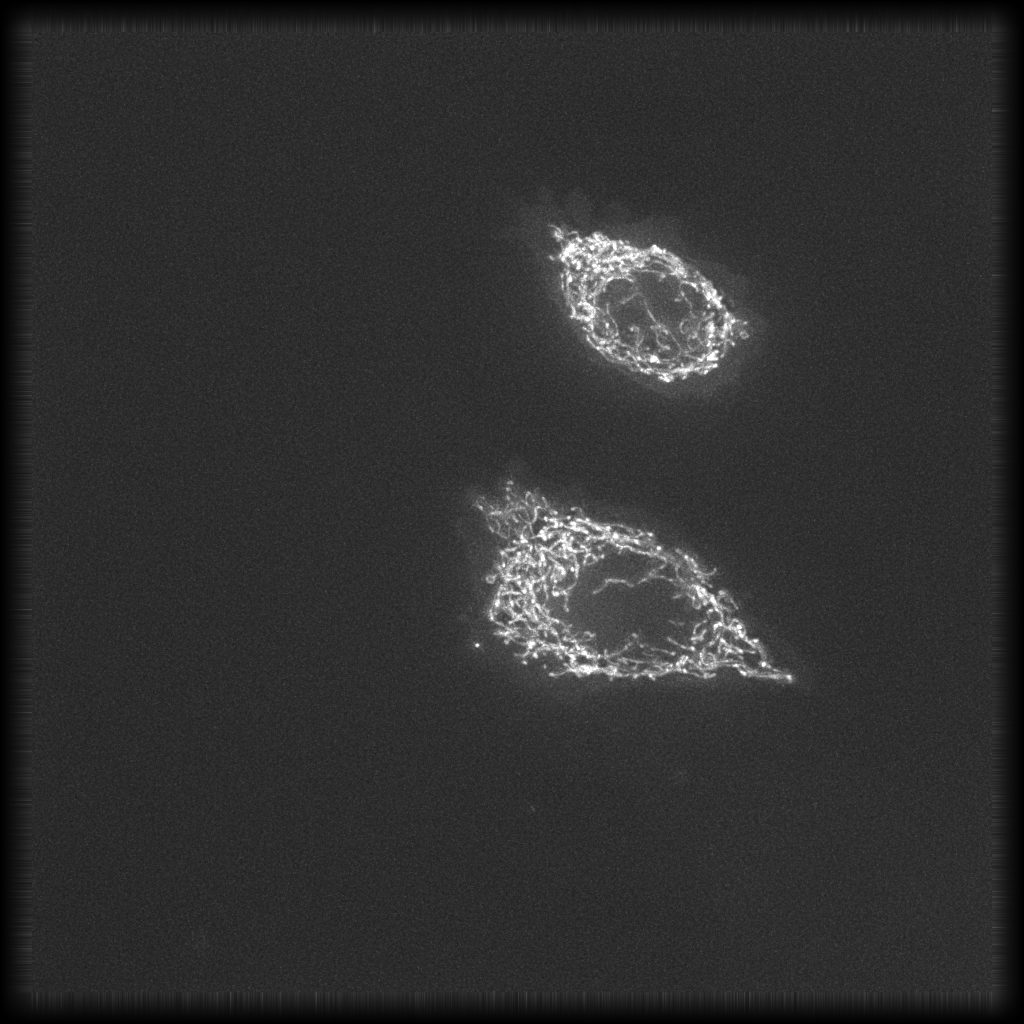

Supplement: Supplementary file 13 — Figure EV3 Source Data [file 44319_2026_776_MOESM13_ESM.zip › FigureEV3/FigureEV3D.tif]

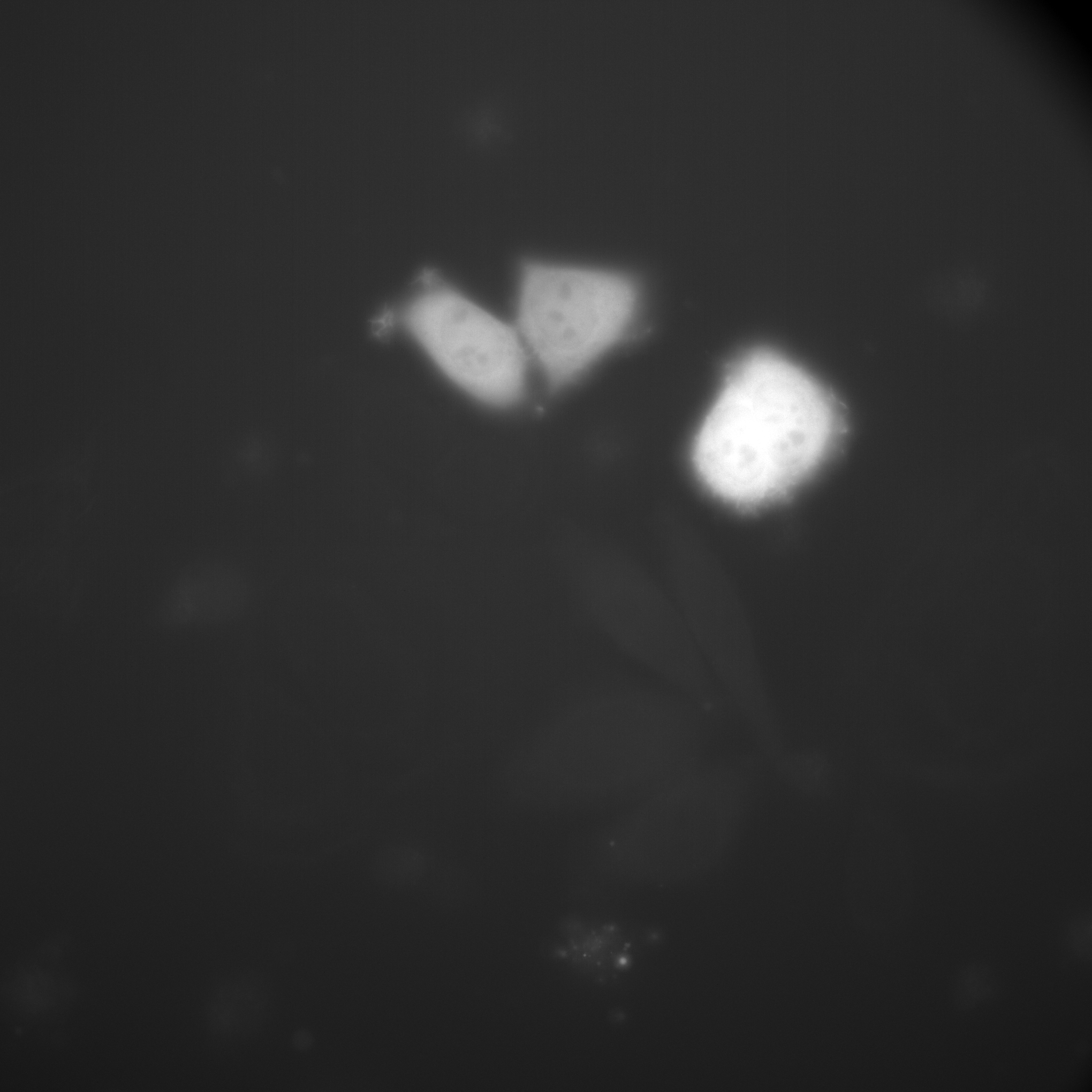

Supplement: Supplementary file 14 — Figure EV5 Source Data [file 44319_2026_776_MOESM14_ESM.zip › FigureEV5/FigureEV5E_bottom.tif]

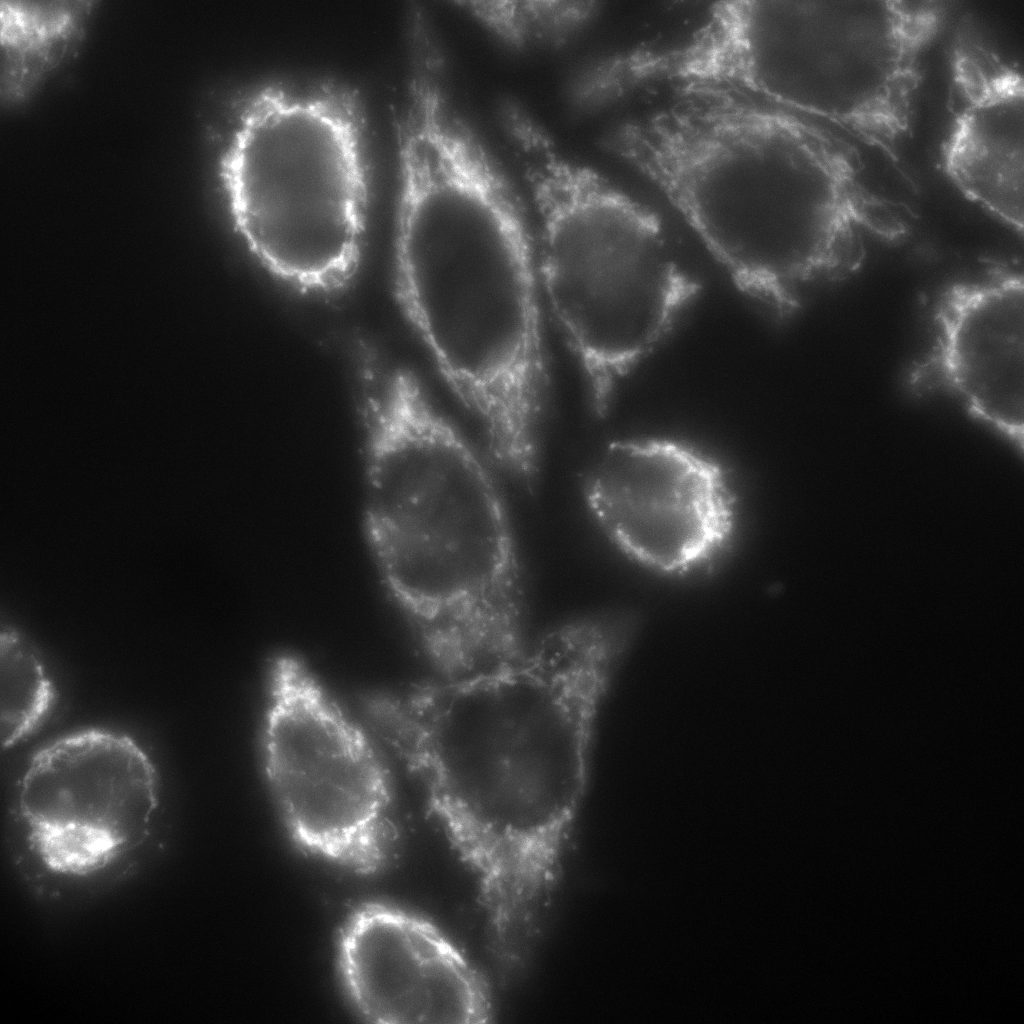

Supplement: Supplementary file 14 — Figure EV5 Source Data [file 44319_2026_776_MOESM14_ESM.zip › FigureEV5/FigureEV5B_left.tif]

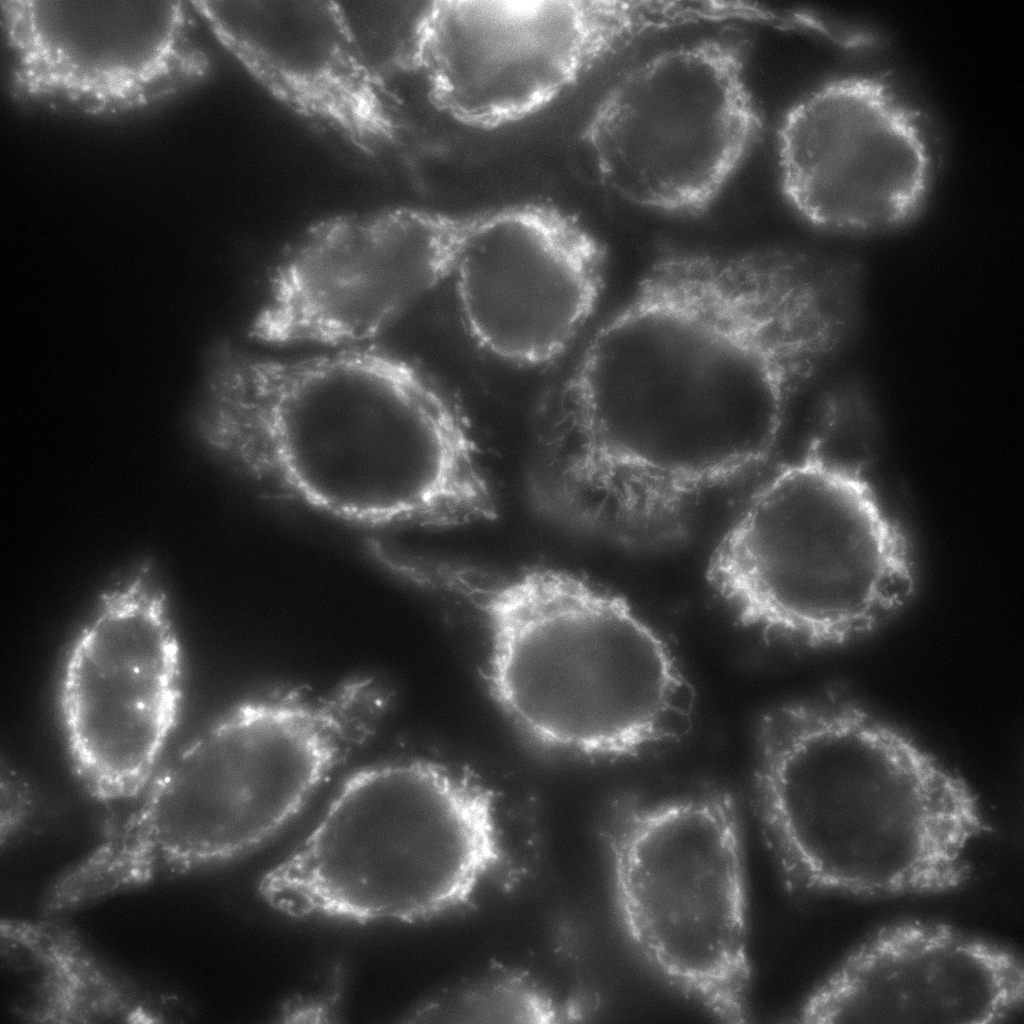

Supplement: Supplementary file 14 — Figure EV5 Source Data [file 44319_2026_776_MOESM14_ESM.zip › FigureEV5/FigureEV5B_right.tif]

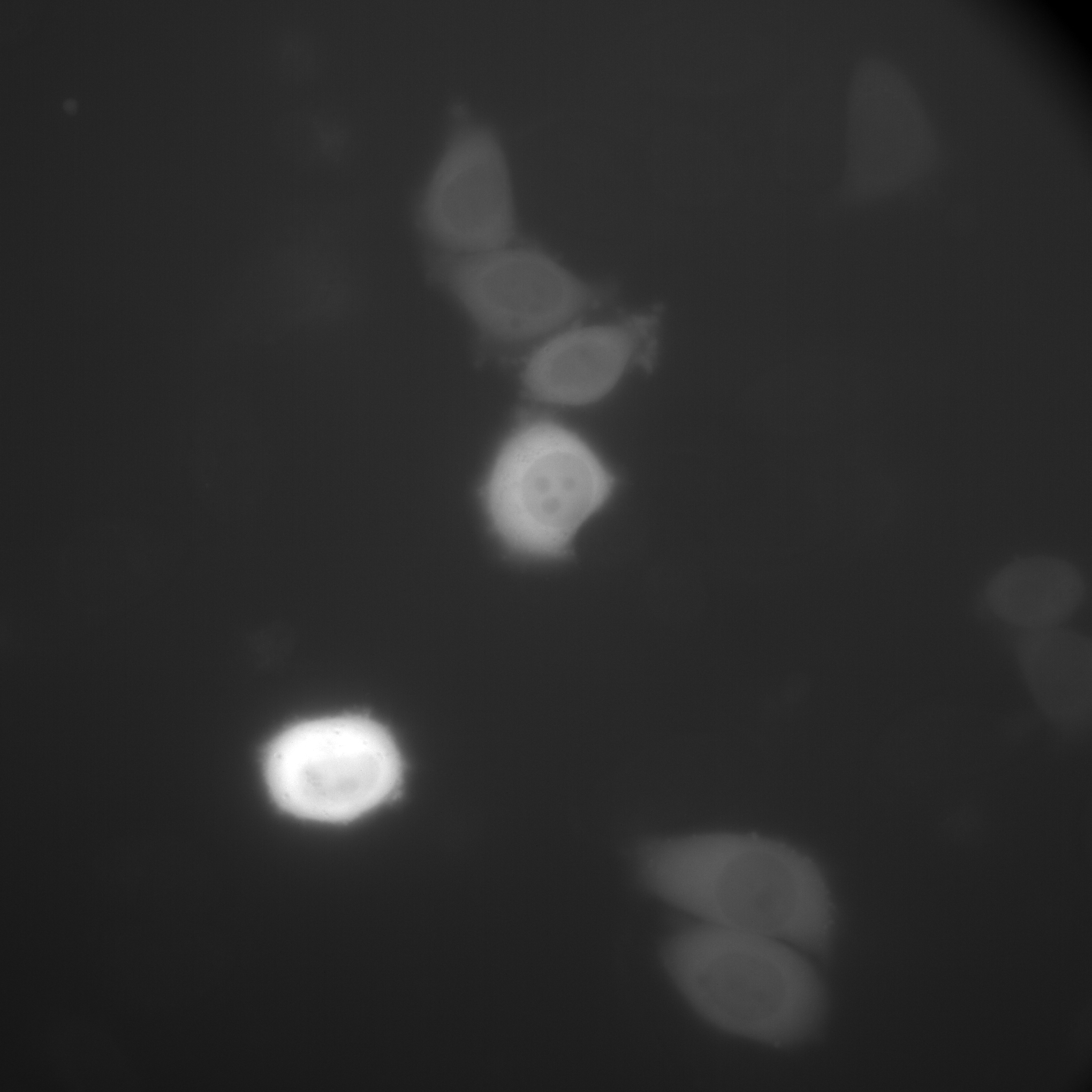

Supplement: Supplementary file 14 — Figure EV5 Source Data [file 44319_2026_776_MOESM14_ESM.zip › FigureEV5/FigureEV5E_top.tif]
