# Supplementary material for: 5′ UTR length shapes alternative N-terminal protein isoforms across cancers and in rare disease
Source: EMBO Rep. 2026 Apr 13;27(10):2823–43. doi: 10.1038/s44319-026-00776-7 (PMC13219423; doi:10.1038/s44319-026-00776-7)
Supplement: Supplementary file 15 — Expanded View Figures [file 44319_2026_776_MOESM15_ESM.pdf]

Expanded View Figures

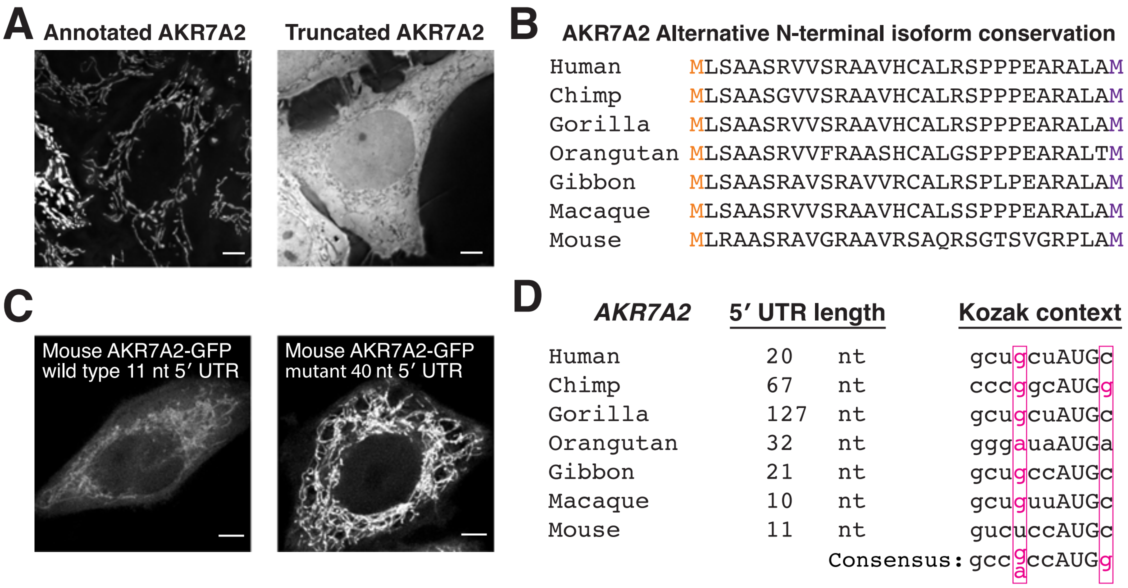

Figure EV1. Evolutionary analysis of AKR7A2 alternative isoforms.

(**A**) Live imaging of annotated human AKR7A2 and truncated AKR7A2 isoforms. (**B**) Protein sequence alignment of AKR7A2 from the indicated mammals. The orange M indicates the annotated start site, and the purple M represents the alternative start site. (**C**) Live imaging of wild-type mouse AKR7A2 and a mutant AKR7A2 mRNA with a longer 5′ UTR. Scale bar indicates 5 μm. (**D**) 5′ UTR length and Kozak context of the first start codon of AKR7A2 in the indicated mammals. Source data are available online for this figure.

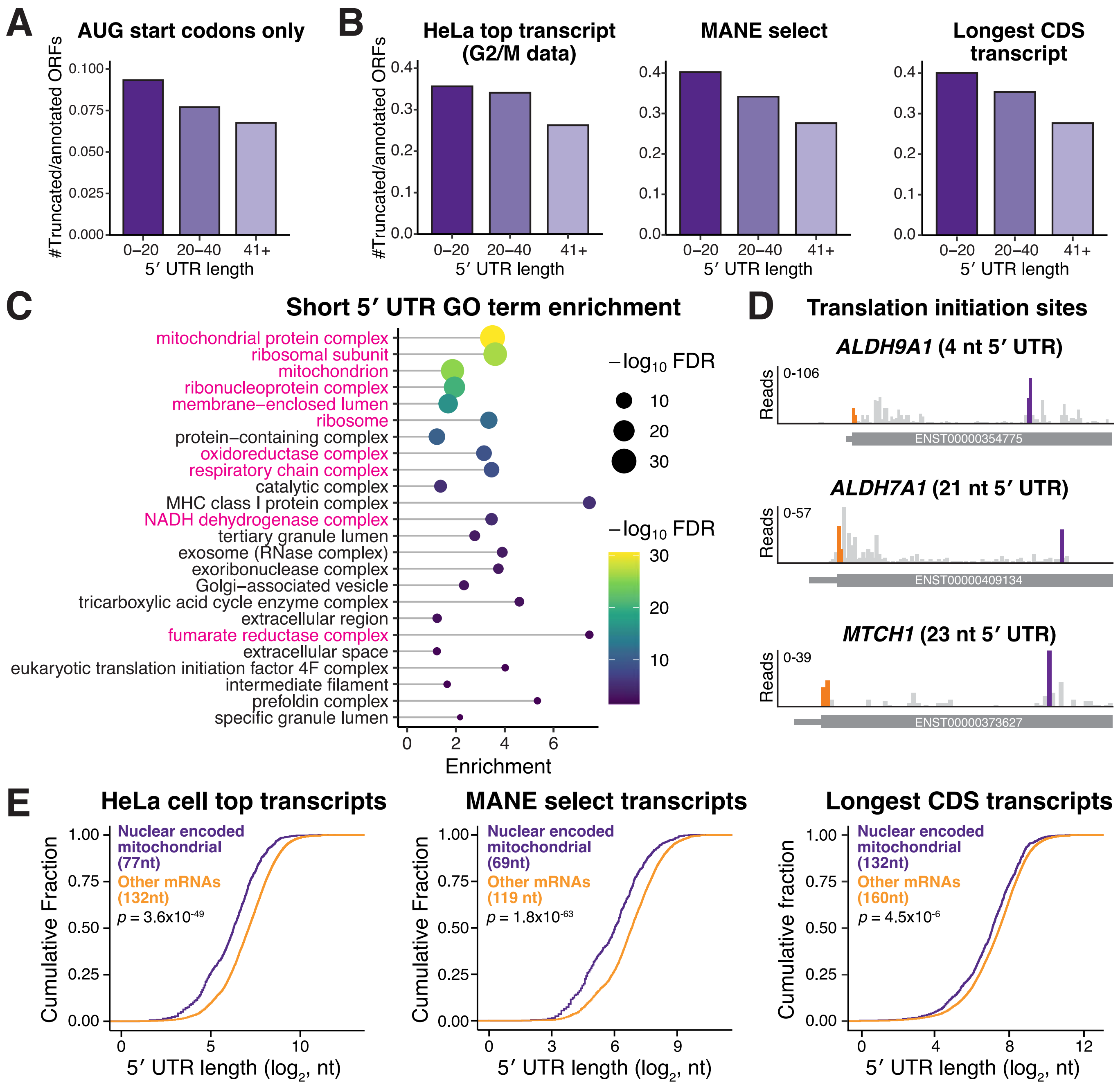

Figure EV2. Relationship between short 5′ UTRs and N-terminally truncated protein isoforms.

(**A**) Same as Fig. 2A except only AUG start codons are included in the analysis. (**B**) Same as Fig. 2A except the representative transcript isoform from which the 5′ UTR length was calculated was from different datasets ("Methods"). (**C**) GO term enrichment analysis of short 5′ UTR mRNAs (≤ 40 nt) from the MANE select transcripts (rather than the highest expressed HeLa transcript in Fig. 2B) compared to transcripts with 5′ UTR lengths >40 nt. Pink GO terms indicate mitochondrial-associated terms. (**D**) Translation initiation site profiling traces around the start codons of indicated mRNAs. (**E**) CDF plot as described in Fig. 2C, except the representative transcript isoform for each gene was selected by different strategies ("Methods"). Statistics indicate Wilcoxon rank-sum test. Source data are available online for this figure.

 © The Author(s)

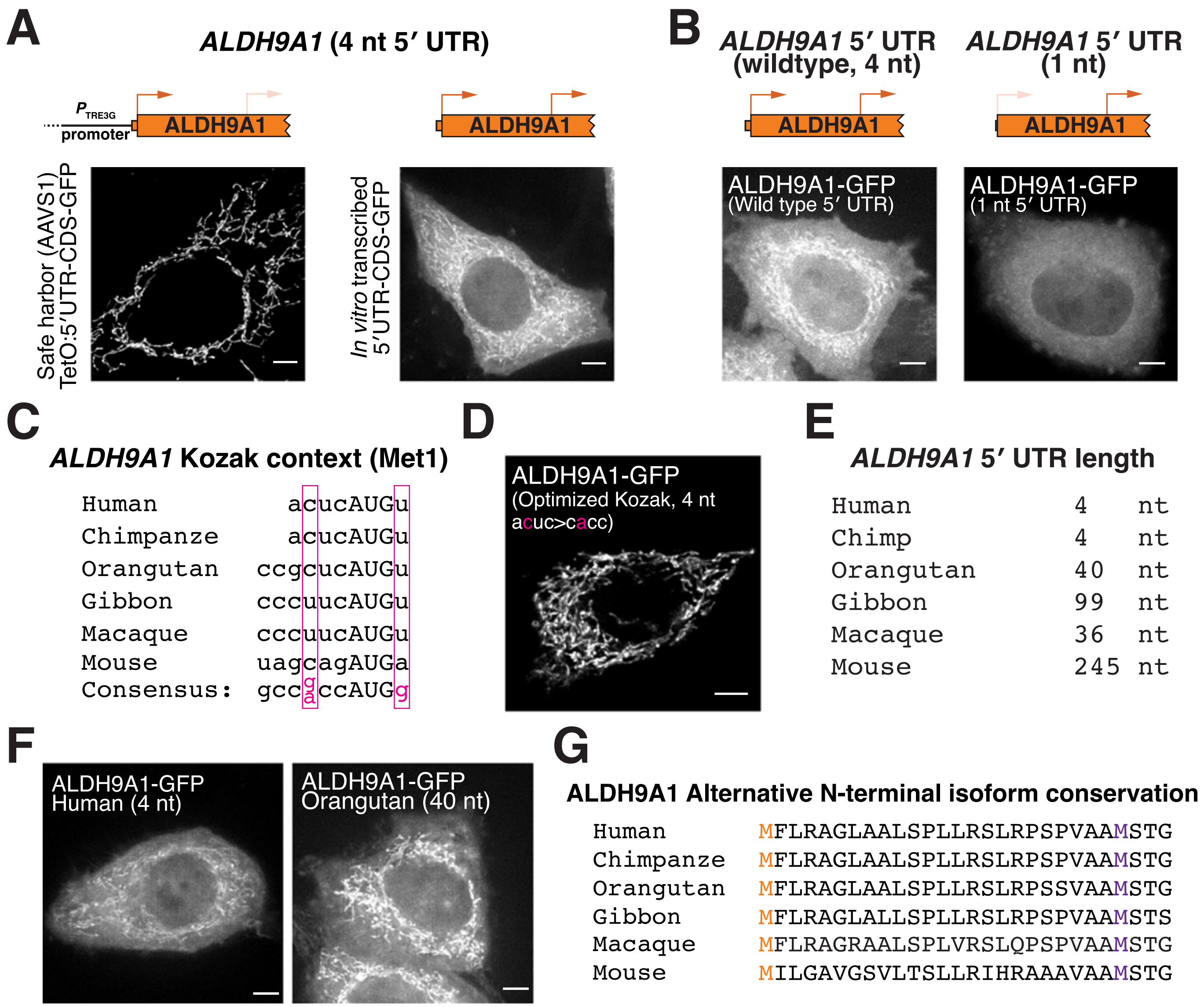

Figure EV3. Additional analysis of ALDH9A1 start codon selection.

(**A**) Live-cell imaging of ALDH9A1-GFP produced from dox-inducible promoter or by transfected in vitro-transcribed mRNA. (**B**) Live-cell imaging of transfected in vitro-transcribed ALDH9A1-GFP with the wild-type (4 nt) or shorter (1 nt) 5′ UTR. (**C**) Conservation of weak Kozak context around the first AUG in ALDH9A1 across selected mammals. (**D**) Live-cell imaging of transfected in vitro-transcribed ALDH9A1-GFP with the optimized Kozak context. (**E**) Analysis of ALDH9A1 5′ UTR lengths across organisms. (**F**) Live-cell imaging of in vitro-transcribed human or orangutan ALDH9A1-GFP. Scale bar indicates 5 µm. (**G**) Protein alignment for ALDH9A1 in the indicated organisms. The orange M indicates the annotated start site, and the purple M represents the alternative start site. Source data are available online for this figure.

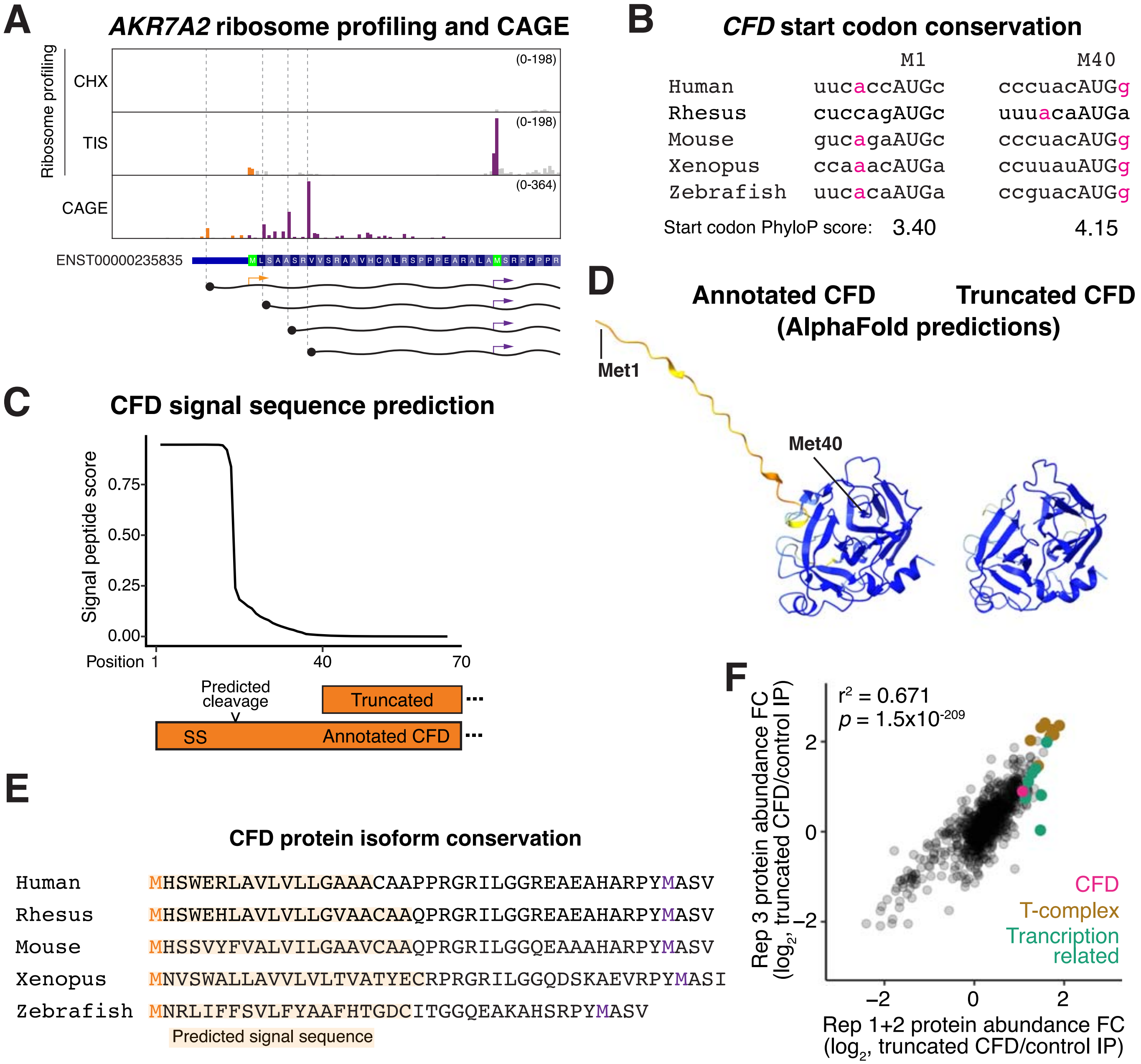

Figure EV4. Additional analysis of alternative AKR7A2 and CFD isoform.

(**A**) Read distribution traces from ribosome profiling and CAGE-seq reveals alternative promoter for AKR7A2 that removes the first AUG. (**B**) Conservation of the CFD start codon across selected organisms. (**C**) CFD SignalP predicts a strong N-terminal secretion signal. (**D**) AlphaFold (Abramson et al, 2024) prediction of annotated and truncated CFD isoform. (**E**) CFD protein sequence alignment. The orange share represents the predicted signal sequence based on SignalP. The orange M indicates the annotated start site, and the purple M represents the alternative start site. (**F**) Scatterplot of proteins identified in truncated CFD IP-MS across different biological replicates and batches. Statistics indicate *t* test for Pearson correlation coefficient.

 © The Author(s)

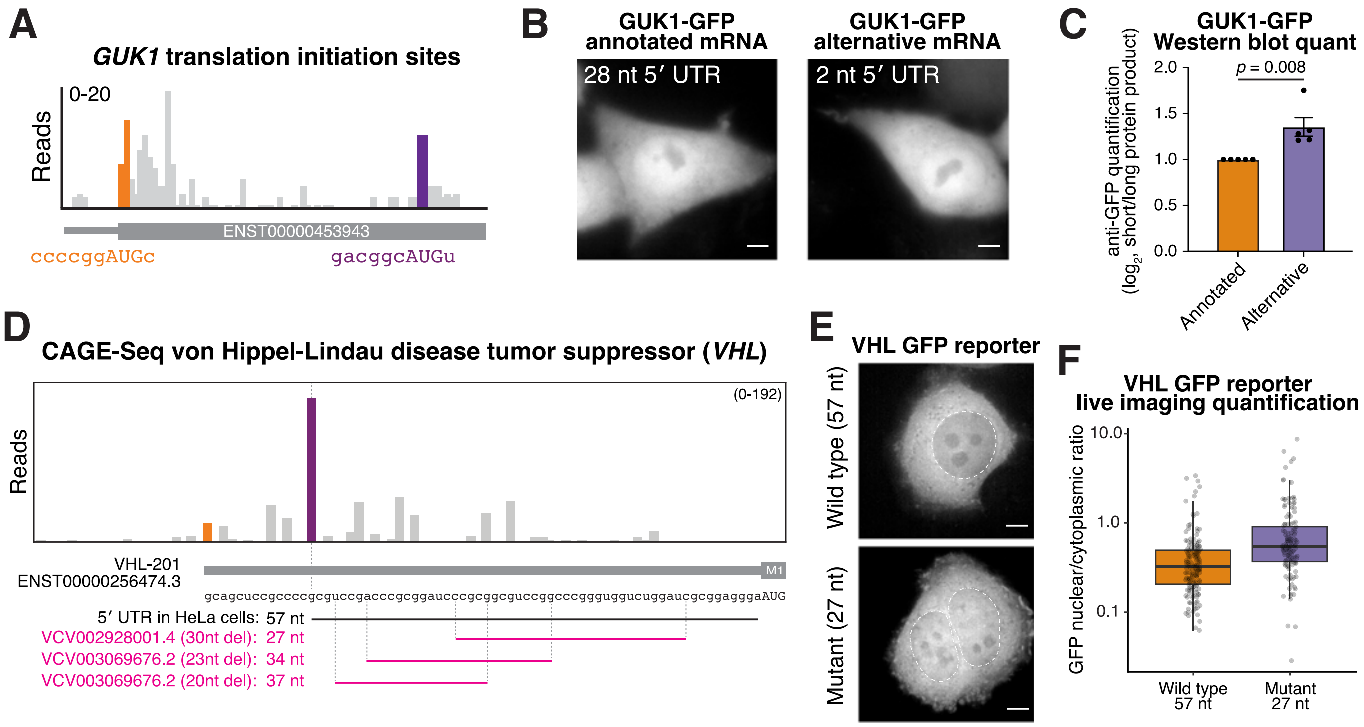

Figure EV5. Additional analysis of GUK1 isoforms.

(**A**) Translation initiation site traces around the start codon for GUK1. (**B**) Live imaging of transfected annotated or alternative GUK1 mRNA with a C-terminal GFP. Images are not scale equally. Scale bar represents 5 μm. (**C**) Quantification of Western blot from Fig. 4F. Error bars indicate standard error of the mean. $n = 5$ biological replicates. Statistics indicate unpaired Student's T test. (**D**) CAGE-seq trace from HeLa cells highlighting that HeLa cells have a shorter 5′ UTR than the annotated isoform. Magenta text and lines represent ClinVar deletions. (**E**) Representative live imaging of HeLa cells transfected with the indicated in vitro-transcribed reporters. Scale bar indicates 5 μm. (**F**) Quantification of nuclear vs cytoplasmic ratio of the VHL GFP reporters. Each point represents a single cell. $n = 2$ biological replicates. Boxplot represents 25th, 50th, and 75th percentile, and whiskers indicate 1.5× interquartile range. Source data are available online for this figure.

© The Author(s)
